# Increasing the steric hindrance around the catalytic core of a self-assembled imine-based non-heme iron catalyst for C–H oxidation†

**DOI:** 10.1039/d0ra09677f

**Published:** 2020-12-24

**Authors:** Federico Frateloreto, Giorgio Capocasa, Giorgio Olivo, Karim Abdel Hady, Carla Sappino, Marika Di Berto Mancini, Stefano Levi Mortera, Osvaldo Lanzalunga, Stefano Di Stefano

**Affiliations:** Dipartimento di Chimica and Istituto CNR per i Sistemi Biologici (ISB-CNR), Sezione Meccanismi di Reazione, Università di Roma La Sapienza P. le A. Moro 5 00185 Rome Italy stefano.distefano@uniroma1.it; Area of Genetics and Rare Diseases, Unit of Human Microbiome, Bambino Gesù Children's Italy

## Abstract

Sterically hindered imine-based non-heme complexes 4 and 5 rapidly self-assemble in acetonitrile at 25 °C, when the corresponding building blocks are added in solution in the proper ratios. Such complexes are investigated as catalysts for the H_2_O_2_ oxidation of a series of substrates in order to ascertain the role and the importance of the ligand steric hindrance on the action of the catalytic core 1, previously shown to be an efficient catalyst for aliphatic and aromatic C–H bond oxidation. The study reveals a modest dependence of the output of the oxidation reactions on the presence of bulky substituents in the backbone of the catalyst, both in terms of activity and selectivity. This result supports a previously hypothesized catalytic mechanism, which is based on the hemi-lability of the metal complex. In the active form of the catalyst, one of the pyridine arms temporarily leaves the iron centre, freeing up a lot of room for the access of the substrate.

## Abstract 2

A case in which the insertion of large and hindering groups in the catalyst backbone does not cause the insurgence of steric effects on the catalysed reaction.

## Graphical abstract

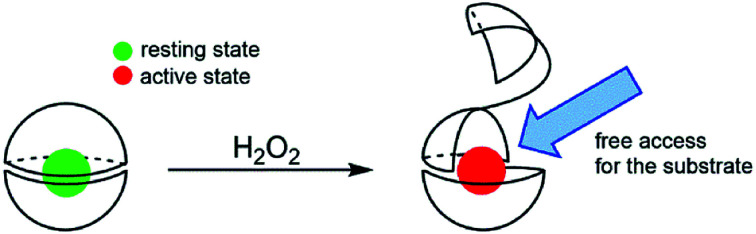

## Introduction

C–H functionalization is a prominent topic in contemporary research due to the opportunity of a late stage derivatization of organic compounds. The chance of a direct installation of a heteroatom on non-activated positions has opened new synthetic routes and methodologies.^1^ In particular, our attention was ultimately oriented to the sustainable oxygenation of aliphatic and aromatic C–H bonds carried out with green terminal oxidants such as hydrogen peroxide (H_2_O_2_) in the presence of non-heme iron complexes^2^ based on the imine function.^3,4^ The choice of this kind of catalyst was guided by a series of needs: (i) use of an abundant and environmentally friendly metal, (ii) easy and (iii) cheap preparation of the catalyst.

**Scheme 1:**
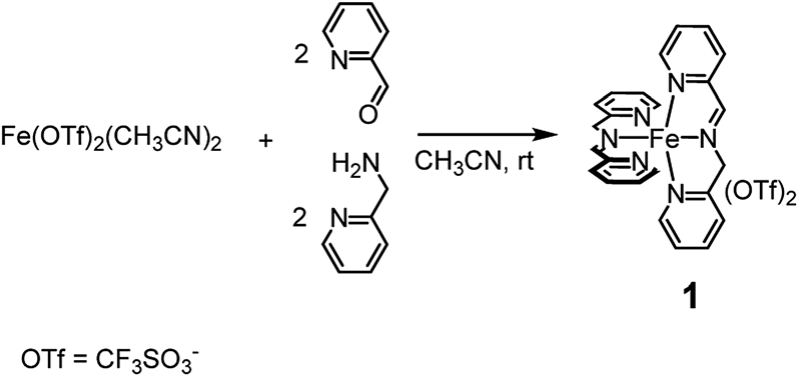
*In situ* preparation of complex 1 from cheap and commercially available precursors. Within 5 min from the addition of the precursors the complex is completely formed and the solution becomes deep violet.

Complex 1, which can be assembled in acetonitrile by simple addition of the cheap and commercially available Fe(CF_3_SO_3_)_2_, 2-picolylaldehyde and 2-picolylamine added in a 1 : 2 : 2 ratio, respectively (see Scheme 1), was demonstrated to satisfy the above requirements. In a number of reports it was shown that complex 1 competes on equal terms with many of the most popular amine-based non-heme iron catalysts^5^ appearing in the literature so far, both in the oxidation of aliphatic and aromatic C–H bonds.

The reaction mechanism adopted by complex 1 in the H_2_O_2_ oxidation of both aliphatic and aromatic substrates was deeply studied^2*b*,*d*^ but some features remain still poorly understood mostly due to the elusive character of the involved active species. The hemi-lability of at least one of the pyridine arms on the metal centre is believed to be essential for the iron activation of the H_2_O_2_, however, while the metal-based nature of the active species is given for certain, its precise structure is still unknown. An iron five oxo-complex N_5_Fe^V^=O was hypothesized^2*b*,*d*^ although an iron four (N_5_Fe^IV^=O) based pathway cannot be excluded.^6^

**Fig. 1:**
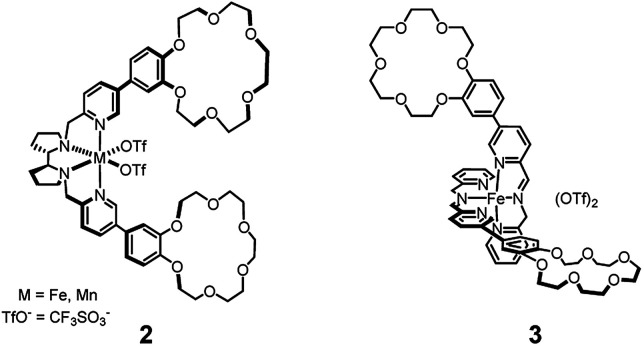
Catalysts endowed with crown-ether receptors for substrate decorated with ammonium head. Catalyst 2 is based on the M(pdp) catalytic core (amino-pyridine catalyst) while catalyst 3 on complex 1 (imino-pyridine catalyst).

In the recent literature supramolecular, non-covalent interactions have been employed in order to direct the selective oxidation of particular C–H bonds among many others present in the same substrate.^7^ In one case^7*a*–*c*^ two crown-ether receptors were implanted in the ligand of a catalyst based on the Mn or Fe(pdp) core,^1*i*,5*b*^ for the recognition of an ammonium anchoring group^8^ in the substrate (see Fig. 1, complex 2). Recognition of the ammonium head of the substrate by the crown-ether receptors allowed the selective oxidation of C–H bonds located at the right distance from the anchoring group (8–9 simple bonds). Application of the same strategy to the imine-based catalytic core 1 (see Fig. 1, complex 3), did not afford comparable results in terms of selectivity.^9^ In the latter case, the difference between the selectivity properties of complexes 1 and 3, devoid of and endowed with the crown-ether receptors, respectively, towards the oxidation of the C–H bonds present in the tested substrates was not evident enough, and when appreciable, it was ascribed to a mere steric hindrance rather than to recognition by the crown-ether moieties. In fact, steric hindrance around the catalyst is known to affect the reaction selectivity, favouring oxygenation of the most accessible sites (C–H or C=C bonds) on the substrate.^1*k*,10,11^

In order to shed light on the role and importance of steric hindrance on the action of catalyst 1 we investigated in detail the effect of the presence of bulky substituents in the catalyst structure. Here below we report the results of such investigation.

## Results and discussion

**Scheme 2:**
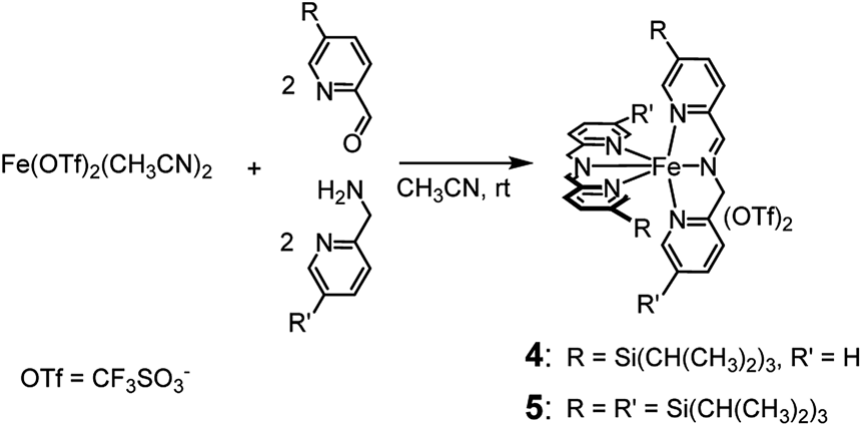
*In situ* preparation of complexes 4 and 5. Within 45 and 75 min, respectively, from the addition of the precursors, the complexes are completely formed and the solution becomes deep violet.

Like complexes 1 and 3, new complexes 4 and 5, which are characterized by an increased steric hindrance around the catalytic core due to the presence of triisopropylsilyl (TIPS) groups, were prepared *in situ* by self-assembly of the parent compounds added in solution in the proper ratios (see Scheme 2), in acetonitrile at 25 °C.

^1^H NMR monitoring of the related solutions showed that complexes 4 (see ESI, Fig. S6†) and 5 (Fig. S18†) are completely formed within 45 min and 75 min, respectively, in contrast with complex 3, whose assembly requires 60 h at the same temperature. Formation of complex 1 under the same conditions is completed in 5 min.^2*a*^ Characterization of the new complexes, based on ^1^H and ^13^C NMR monitoring, HSQC 2D NMR, HR-MS and UV-Vis spectroscopy, showed the high similarity of complexes 4 and 5 with the parent complex 1 (compare, for example, trace at *t* = 44 min in Fig. S6† with the ^1^H NMR spectrum of complex 1 reported in Fig. S1†). The UV-Vis spectra reported in Fig. S22† show the near resemblance among complexes 1, 4 and 5. Apart from a modest bathochromic effect due to the TIPS groups, the shape of the bands related to the iron core from 400 to 700 nm remains practically unchanged. Eventually, the 2 : 2 : 1 stoichiometry of the new complexes 4 and 5 is definitely demonstrated by the Job's plots reported in the ESI (Fig. S11 and S21,† respectively). Thus, the only difference among complexes 1, 4 and 5 should lie in the increasingly limited access to the metal centre.

The results obtained in the H_2_O_2_ oxidation of a series of aromatic compounds carried out in the presence of complexes 4 and 5 are reported in Table 1 together with those obtained with catalyst 1 under the same conditions for the sake of comparison. In all cases hardly detectable trace amounts or no trace of products with oxidized lateral chain were found, in accordance with the well-known preference of this imine based catalytic core for aromatic C–H oxidation with respect to aliphatic C–H oxidation.^2*d*^

**Table 1:** H_2_O_2_ Oxidation of ethylbenzene, cumene and *tert*-butylbenzene carried out in acetonitrile at room temperature in the presence of catalysts 1, 4 and 5^a^^,^^b^

Cat.	Substrate^c^ (recov.%)	*ortho* c (yield%)	*meta* + *para*^c^ (yield%)	(*m* + *p*)/*o*^d^
	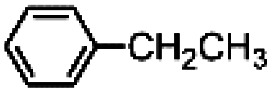	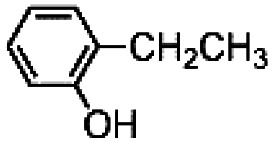	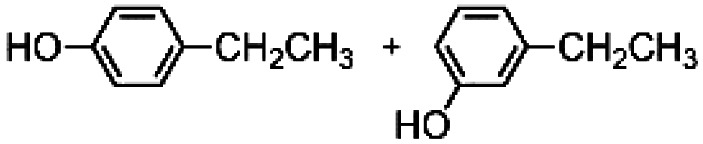	
1	53_7_	7.1_0.1_	8.5_0.1_	1.20_0.02_
4	52_4_	7.2_0.1_	8.3_0.1_	1.10_0.04_
5	63_9_	7.7_0.9_	7.6_0.9_	0.98_0.22_
	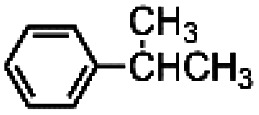	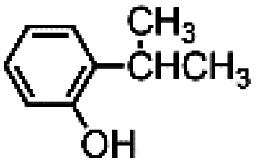	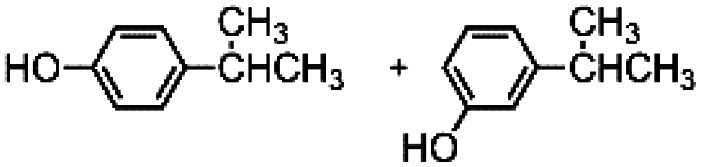	
1	69_9_	8.1_0.9_	16_2_	2_0.5_
4	64_9_	7.4_0.3_	13_1_	1.8_0.3_
5	74_3_	7.4_0.1_	11_1_	1.5_0.1_
	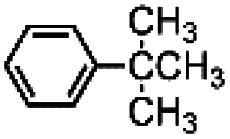	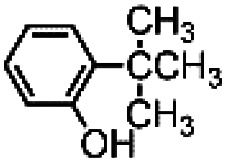	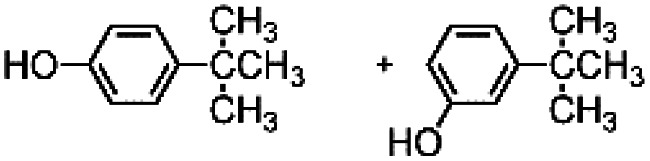	
1	50_1_	8.2_1_	18_1_	2.2_0.4_
4	58_2_	7.9_0.1_	17_1_	2.2_0.1_
5	60_1_	8.0_0.3_	16_1_	2.0_0.2_

a Reaction conditions are: substrate 0.20 M, H_2_O_2_ 2.5 mol eq. added in 30 min with a syringe pump, catalyst 2% mol, 25 °C, acetonitrile/H_2_O, 1 h 30 min total reaction time.

b Yields from GC measurements calculated using nitrobenzene (0.5 mol eq.) as the internal standard.

c Error is calculated from at least three independent runs.

d Error from propagation applied to at least three independent runs.

When catalyst 1 is taken into account, total yield of oxidation generally increases on increasing the size of the lateral alkyl chain of the substrate as previously observed.^2*d*^ When complexes 4 and 5 are used as catalysts under the same conditions, quite astonishingly and in contrast with what was found with catalyst 3,^9^ the yields of phenol products remain more or less the same for each substrate in comparison with catalyst 1 in the limit of experimental errors. Even more surprising is the fact that the presence of two or four, very bulky TIPS groups in the catalyst backbone does not influence at any extent the (*meta* + *para*)/*ortho* ratio in the reaction products. Such ratio remains the same along each series 1, 4 and 5.

Although the exact nature of the active species involved in the reaction is still unknown, a series of clues collected in previous investigations^2^ prompted us to hypothesize a mechanism in which, after an initial outer-sphere oxidation of Fe^II^ to Fe^III^, the temporary detachment of one of the pyridine arms would unmask the iron centre, which could be available to take part to the oxidation process, generating an initial Fe^III^–OOH based species (Fig. S23†).

A possible evolution of the latter could be the heterolytic cleavage of the peroxidic bond leading to a Fe^V^=O species competent for the oxidative properties of the system.^5*a*–*d*^ On the other hand, the homolytic cleavage of the same bond would originate an in-cage couple {Fe^IV^=O + HO˙}. The HO˙ radical would then attack the aromatic ring leading to a hydroxycyclohexadienyl radical, which in turn, would be oxidized by Fe^IV^=O to give the product upon deprotonation and to restore the Fe^III^ species.^6^ However, whichever the active species is, many pieces of evidence have been collected pointing to a controlled oxidation mechanism where free radical oxidations do not play any role.^2^

The fact that in the series reported in Table 1 the (*meta* + *para*)/*ortho* selectivity remains practically untouched for each substrate no matter what the catalyst is, is a clear-cut proof of the absence of any steric interaction between the alkyl chain of the substrate and the TIPS groups mounted on the ligand. A little but appreciable substrate steric effect can be noted in the series ethylbenzene, cumene, *tert*-butylbenzene. Independently of the nature of the catalyst the (*meta* + *para*)/*ortho* ratio steadily increases along the series (1, 1.5, 2, respectively), however the access to the active core must be the same for all of three catalysts 1, 4 and 5.

**Fig. 2:**
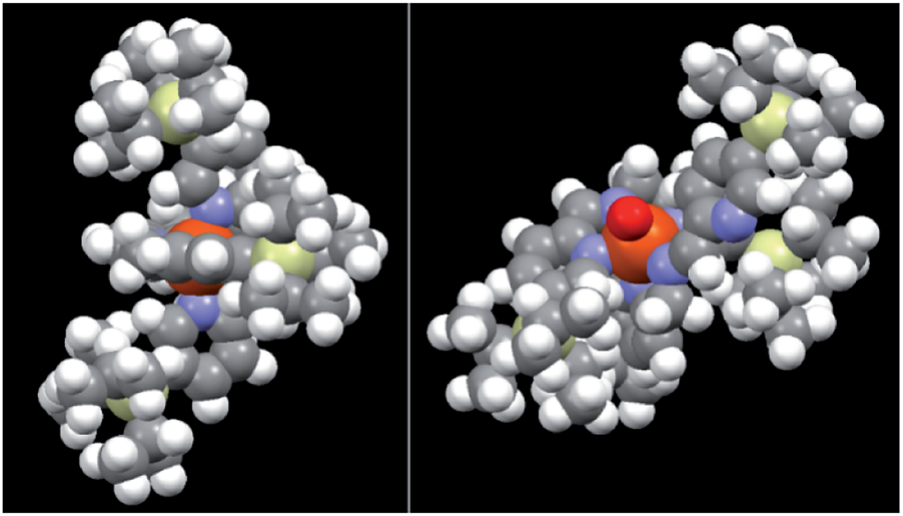
CPK models of complex 5 in its resting state (Fe^II^, left) and in its putative active form (Fe^V^=O, right).

A possible explanation for the above, quite unexpected results is that, although the catalytic core of 4 and particularly of 5 appears to be highly hindered in the close form (Fig. 2, left, CPK model of complex 5), when one of the pyridine arms is opened by H_2_O_2_ (see Fig. 2 right where a CPK model of the open Fe^V^=O putative active species is shown), the steric hindrance offered by the TIPS group decreases in such an extent as to become completely unimportant when the monosubstituted substrates reported in Table 1 are considered.

**Fig. 3:**
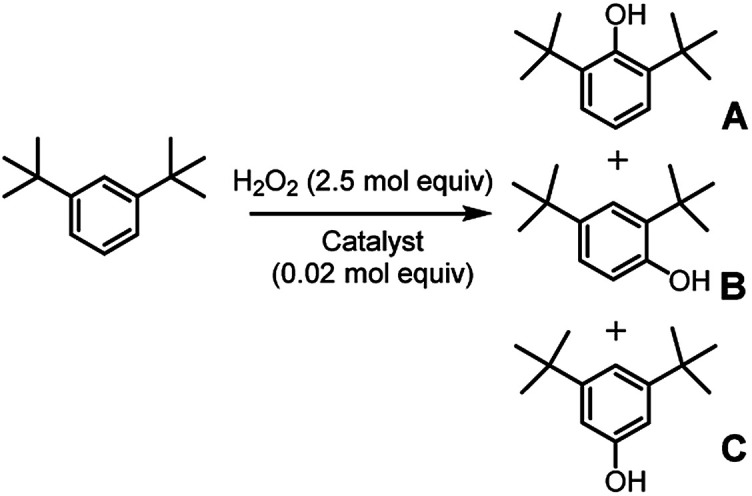
Oxidation of 1,3-di-*tert*-butylbenzene. The reported yields derive from GC analysis of the reaction crude after work-up and addition of nitrobenzene (0.5 mol eq.) as the internal standard. The oxidant was added over the course of 30 minutes, after which the mixture was allowed to react for 1 hour at 25 °C.

In an attempt to exasperate the steric hindrance of the substrate, 1,3-di-*tert*-butylbenzene (Fig. 3) was employed in the study and oxidized in the presence of complex 1 and complex 5 which are the extreme limits of the series. In this case, with both catalysts phenol B was found to be the largely predominant product, with trace amounts of C and no trace of A. However, also in this case yield of phenol B, remains the same for catalysts 1 and 5, 7.0 ± 0.3% and 7.3 ± 0.1%, respectively. It has to be mentioned that under the same conditions but in the absence of any ligand (Fenton-like conditions), although the total yield was extremely low, also phenol A was found in the reaction crude.

Thus, to sum up, in the case of oxidation of simple aromatic substrates, we were not able to detect any effects due to the presence of the TIPS groups on the backbone of the catalyst.

**Fig. 4:**
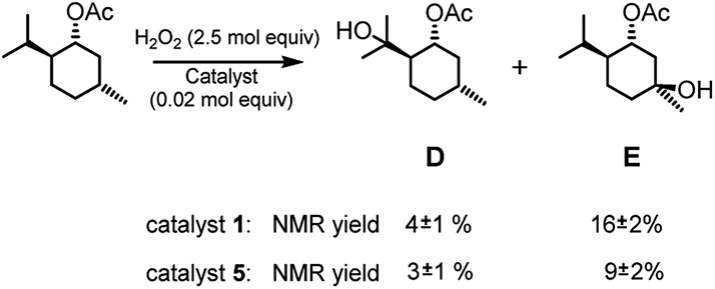
Oxidation of menthyl acetate. The reported yields are obtained by ^1^H NMR analysis of the reaction crude after work-up and addition of bibenzyl (0.5 mol eq.) as the internal standard. The oxidant was added over the course of 30 minutes, after which the mixture was allowed to react for 1 hour at 25 °C.

Given that complex 1 is also known to be active in the catalysis of the oxidation of aliphatic C–H bonds,^2*a*,*b*^ complexes 1 and 5 were also compared in the oxidation of menthyl acetate (see Fig. 4). Menthyl acetate is indeed often used as a benchmark substrate in order to evaluate steric effects on the regioselectivity of C–H oxidation. In both cases alcohols D and E were the major products. In the case of catalyst 1 total yield D + E was about 20% with a D/E ratio of 0.25. When catalyst 5 was used, total yield D + E was about 12% with a D/E ratio of 0.33. Thus, it is evident that for this substrate the steric effect of the TIPS groups is not unimportant and the D/E selectivity increases on increasing the steric hindrance on the catalyst, the peripheral isopropylic C–H bond being sterically more accessible than the tertiary endocyclic one.

**Fig. 5:**
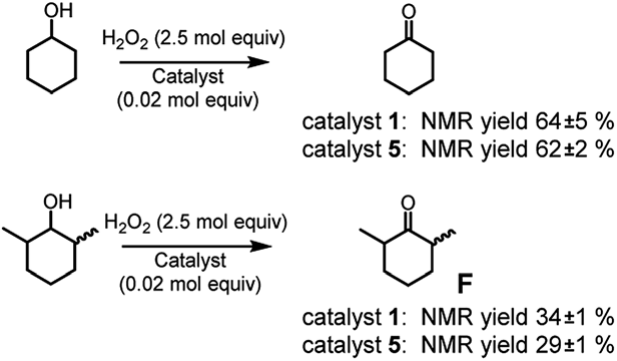
Oxidation of cyclohexanol and 2,6-dimethylcyclohexanol. The reported yields are obtained by ^1^H NMR analysis of the reaction crude after work-up and addition of bibenzyl (0.5 mol eq.) as the internal standard. The oxidant was added over the course of 30 minutes, after which the mixture was allowed to react for 1 hour at 25 °C.

Eventually, since complex 1 was proved to be an active catalyst also for alcohol oxidation^2*c*^ (although this reaction cannot be properly considered a C–H oxidation but more properly an oxidation, from a steric standpoint it can serve to our purpose), we tested complexes 1 and 5 in the oxidation of cyclohexanol and its more sterically congested analogue 2,6-dimethylcyclohexanol (*cis* + *trans* mixture, see Fig. 5) to cyclohexanone and 2,6-dimethylcyclohexanone F, respectively.

While no difference appears in the oxidation of cyclohexanol, complex 5 seems to be less active in the oxidation of 2,6-dimethylcyclohexanol with respect to complex 1. The effect is modest, but the origin of such difference has to be steric in nature. It should be noted that catalyst 1 was already found to be quite sensitive to steric effects in the oxidation of α-(poly)substituted cyclohexanols.^2*c*^ Thus, the increased volume of complex 5, seems to moderately enhance the catalyst sensitivity to the steric bulk of the substrate.

## Conclusions

In this report the synthesis of new complexes 4 and 5 is described. Self-assembly of both complexes is a relatively fast process taking place within 45 and 75 min, respectively, in CH_3_CN at 25 °C when the starting building blocks are added in solution in the proper ratios. Complexes 4 and 5 essentially differ from the parent complex for an increased steric hindrance around the metal ion.

When complexes 4 and 5 were tested as catalysts for the H_2_O_2_ oxidation of a series of monoalkyl-substituted aromatic compounds, their catalytic activity was found to be nearly superimposable to that of the parent complex 1, within the experimental error. This behaviour was ascribed to a strong decrease of the steric hindrance when passing from the resting state to the active state of the catalyst, where one of the pyridine arms has temporarily left the iron centre, freeing up space for the access of the substrate. The absence of any steric effect was also found in the oxidation of a disubstituted aromatic substrate like 1,3-di-*tert*-butylbenzene.

Although moderate, appreciable differences between the efficiencies of catalysts 1 and 5 in term of yields and selectivity instead arise when substrates endowed with hindering groups around non-aromatic C–H oxidizable bonds are considered.

In conclusion it could be stressed that the insertion of large and hindering groups in the catalyst backbone does not necessary lead to the insurgence of steric effect on the catalysed reaction. The data collected in this report reinforce the mechanistic hypothesis depicted in Fig. S23,† remarking the importance of the hemi-lability of the complex in the explication of the catalytic action.

## Experimental

### Methods and material

GC analyses were carried out on a Varian CP-3800 gas chromatograph equipped with a capillary methylsilicone column (30 m × 0.25 mm × 25 μm) Chrompack CP-Sil 5 CB. NMR spectra were recorded on a Bruker AC300 (300 MHz) spectrometer and were internally referenced to the residual proton solvent signal. HR-MS were acquired with a Thermo Scientific Orbitrap Fusion Mass Spectrometer equipped with an ESI ion source. UV analyses were carried out with HP HEWLETT PACKARD 8453 spectrophotometer. All reagents and solvents were purchased at the highest commercial quality and were used without further purification unless otherwise stated. Fe(CH_3_CN)_2_(OTf)_2_ (^−^OTf = CF_3_SO_3_^−^) was prepared according to a literature procedure^12^ from Fe(ii) chloride (Sigma Aldrich). Solvents were purchased from Sigma Aldrich and used as received.

5-Triisopropylsilyl-pyridine-2-carboxaldehyde^11,13^ and 5-triisopropylsilyl-2-picolylamine^11*b*^ were prepared according to literature procedure and spectral data were in accordance with those reported. Menthyl acetate^14^ and 2,6-dimethylcyclohexanol^15^ were prepared according to literature procedures and spectral data were in accordance with those reported.

### Preparation (self-assembly) of complexes (1.0 × 10^−2^ M stock solutions in CH_3_CN)

#### Complex 1

Was prepared according to the literature procedure^2^ and spectral data were in accordance with those reported.

#### Complex 4

Adapting the literature procedures,^2*d*^ 4.36 mg (1.00 × 10^−5^ mol, 1.0 mol equiv.) of Fe(CH_3_CN)_2_(OTf)_2_ and 100 μL of a 0.200 M acetonitrile solution of 5-triisopropylsilyl-pyridine-2-carboxaldehyde (2.00 × 10^−5^ mol, 2.0 mol equiv.) were placed in a 1 mL volumetric flask. A small amount of acetonitrile was added to fully dissolve the reactants. 100 μL of a 0.200 M acetonitrile solution of 2-picolylamine were added (2.00 × 10^−5^ mol, 2 mol equiv.). The solution turned violet and was brought to 1 mL total volume by addition of acetonitrile. The complex was proved to be completely formed after 45 min. The complex was used without further purification. ^1^H NMR (300 MHz, CD_3_CN): *δ* 10.32 (s, 2H), 8.17 (d, *J* = 7.7 Hz, 2H), 7.98–7.86 (m, 4H), 7.75–7.64 (m, 2H), 7.57 (d, *J* = 7.8 Hz, 2H), 7.23 (s, 2H), 7.08 (dd, *J* = 6.4 Hz, 2H), 6.70 (d, *J* = 23.3 Hz, 2H), 6.46 (d, *J* = 23.4 Hz, 2H), 1.38–1.17 (m, 6H), 1.18–0.91 (m, 6H), 0.87–0.64 (m, 30H). ^13^C NMR (75 MHz, CD_3_CN) *δ* 170.3, 163.0, 158.9, 156.5, 152.2, 144.6, 138.7, 136.5, 127.1, 125.0, 121.5, 62.9, 17.7, 17.4, 17.3, 9.9.‡‡Signals at 17.7, 17.4 and 17.3 ppm for 4 and at 17.6, 17.3 and 17.2 for 5, belong to the CH_3_ on the triisopropylsilyl groups as it can be inferred from the HSQC experiment (see ESI†). The existence of three distinct signals is very likely a consequence of the slowdown of the rotation around the Si–C bond due the increased steric hindrance upon complex formation. HR-MS (ESI/Orbitrap) *m*/*z*: M^2+^: calcd for C_42_H_62_N_6_Si_2_Fe 381.1961; found: 381.1958. *λ*_max_ (CH_3_CN)/nm 494 (*ε*/dm^3^ mol^−1^ cm^−1^ 4600), *λ*_max_ (CH_3_CN)/nm 582 (*ε*/dm^3^ mol^−1^ cm^−1^ 6700).

#### Complex 5

Adapting the literature procedures,^2*d*^ 4.36 mg (1.00 × 10^−5^ mol, 1.0 mol equiv.) of Fe(CH_3_CN)_2_(OTf)_2_ and 100 μL of a 0.200 M acetonitrile solution of 5-triisopropylsilyl-pyridine-2-carboxaldehyde (2.00 × 10^−5^ mol, 2.0 mol equiv.) were placed in a 1 mL volumetric flask. A small amount of acetonitrile was added to fully dissolve the reactants. 100 μL of a 0.200 M acetonitrile solution of 5-triisopropylsilyl-2-picolylamine were added (2.00 × 10^−5^ mol, 2 mol equiv.). The solution turned violet and was brought to 1 mL total volume by addition of acetonitrile. The complex was proved to be completely formed after 75 min. The complex was used without further purification. ^1^H NMR (300 MHz, CD_3_CN) *δ* 10.39 (s, 2H), 8.18 (d, *J* = 7.7 Hz, 2H), 7.93 (dd, *J* = 7.7, 1.3 Hz, 2H), 7.74 (dd, *J* = 7.8, 1.4 Hz, 2H), 7.58 (d, *J* = 8.3 Hz, 4H), 7.30 (s, 2H), 6.63–6.40 (m, 4H), 1.40–1.23 (m, 12H), 1.17–0.96 (m, 20H), 0.87–0.71 (m, 52H). ^13^C NMR (75 MHz, CD_3_CN) *δ* 170.2, 162.6, 158.7, 157.0, 155.7, 145.3, 144.6, 136.7, 132.2, 127.1, 121.0, 62.9, 17.6, 17.3, 17.2, 9.7.‡ HR-MS (ESI/Orbitrap) *m*/*z*: M^2+^: calcd for C_60_H_102_N_6_Si_4_Fe 537.3296; found: 537.3302. *λ*_max_ (CH_3_CN)/nm 498 (*ε*/dm^3^ mol^−1^ cm^−1^ 3900), *λ*_max_ (CH_3_CN)/nm 587 (*ε*/dm^3^ mol^−1^ cm^−1^ 5200).

#### General oxidation protocol for the substrates

Adapting the previous procedure,^2*d*^ 70 μL of a 0.320 M stock solution of substrate (22.4 μmol) were placed in a vial. 44.8 μL of a 1.00 × 10^−2^ M solution of either complex 1, 4 or 5 (0.448 μmol) were added to the vessel and the reaction mixture was thermostatted to 25 °C using a sand bath. 12.5 μL of a 4.48 M acetonitrile solution of H_2_O_2_ (56.0 μmol, 2.5 mol equiv.) were injected into the vial over the course of 30 minutes by means of a syringe pump. After 90 minutes from the start of the addition, the appropriate amount of a stock solution of internal standard (nitrobenzene for GC analysis and bibenzyl for ^1^H NMR, 11.2 μmol, 50% mol equiv. with respect to substrate) was added and the reaction mixture was quickly filtered over a short silica pad eluting with ethyl acetate. The eluate was analysed by GC and the oxidation products were identified by comparison of the retention time with those of authentic compounds,^2*c*,*d*^ and quantified by means of the proper internal standard (see above). In the case of menthyl acetate and the cyclohexane substrates, the oxidation products were identified and quantified by means of ^1^H NMR.^5*d*^ Each run was carried out at least in triplicate.

## Conflicts of interest

There are no conflicts to declare.

## Supplementary Material

RA-011-D0RA09677F-s001

## References

[cit1] Chiappini N. D., Mack J. B. C., Du Bois J. (2018). Angew. Chem., Int. Ed..

[cit2] (f) Ticconi B. , Capocasa G., Cerrato A., Di Stefano S., Lapi A., Marincioni B., Olivo G. and Lanzalunga O., Catal. Sci. Technol., 10.1039/d0cy01868f

[cit3] Ciaccia M., Cacciapaglia R., Mencarelli P., Mandolini L., Di Stefano S. (2013). Chem. Sci..

[cit4] Olivo G., Lanzalunga O., Di Stefano S. (2016). Adv. Synth. Catal..

[cit5] Vicens L., Olivo G., Costas M. (2020). ACS Catal..

[cit6] Thibon A., Jollet V., Ribal C., Sénéchal-David K., Billon L., Sorokin A. B., Banse F. (2012). Chem.–Eur. J..

[cit7] Olivo G., Farinelli G., Barbieri A., Lanzalunga O., Di Stefano S., Costas M. (2017). Angew. Chem., Int. Ed..

[cit8] Di Stefano S., Capocasa G., Mandolini L. (2020). Eur. J. Org. Chem..

[cit9] Capocasa G., Di Berto Mancini M., Frateloreto F., Lanzalunga O., Olivo G., Di Stefano S. (2020). Eur. J. Org. Chem..

[cit10] Milan M., Costas M., Otte M., Klein Gebbink R. J. M. (2017). Adv. Synth. Catal..

[cit11] Font D., Canta M., Milan M., Cussò O., Ribas X., Klein Gebbink R. J. M., Costas M. (2016). Angew. Chem., Int. Ed..

[cit12] Diebold A., Elbouadili A., Hagen K. S. (2000). Inorg. Chem..

[cit13] Cussó O., Cianfanelli M., Ribas X., Klein Gebbink R. J. M., Costas M. (2016). J. Am. Chem. Soc..

[cit14] Álvarez-Calero J. M., Jorge Z. D., Massanet G. M. (2016). Org. Lett..

[cit15] Chowdhury L., Croft C. J., Goel S., Zaman N., Tai A. C. S., Walch E. M., Smith K., Page A., Shea K. M., Hall C. D., Jishkariani D., Pillai G. G., Hall A. C. (2016). J. Pharmacol. Exp. Ther..

