# Supplementary material for: Increasing the steric hindrance around the catalytic core of a self-assembled imine-based non-heme iron catalyst for C–H oxidation
Source: RSC Adv. 2020 Dec 24;11(1):537–42. doi: 10.1039/d0ra09677f (PMC8690968; doi:10.1039/d0ra09677f)
Supplement: RA-011-D0RA09677F-s001 [file RA-011-D0RA09677F-s001.pdf]

Electronic Supplementary Material (ESI) for RSC Advances.
This journal is © The Royal Society of Chemistry 2020

Electronic Supplementary Information

for

Increasing the Steric Hindrance Around the Catalytic Core of a Self-Assembled Imine-Based Non-Heme Iron Catalyst for C-H Oxidation.

Federico Frateloreto,[a] Giorgio Capocasa,[a] Giorgio Olivo,[a] Karim Abdel Hady,[a] Carla Sappino,[a] Marika Di Berto Mancini,[a] Stefano Levi Mortera,[b] Osvaldo Lanzalunga[a] and Stefano Di Stefano,[a]*

Table of contents

Characterization of complex **4** S1
^{1}H NMR spectrum of complex **1** (for comparison)....S1
NMR spectra of complex **4** S2
UV-Vis absorption spectrum of complex **4** S9
Job's plot for complex **4**....S9
Characterization of complex **5** S10
NMR spectra of complex **5** S10
UV-Vis absorption spectrum of complex **5** S17
Job's plot for complex **5**....S17
Cumulative UV-Vis spectra of complexes **1**, **4** and **5** S18
Previously proposed mechanism for activation of H_2O_2 by the imine complex S18

[a]Dipartimento di Chimica, Università degli Studi di Roma "La Sapienza" and Istituto CNR di Metodologie Chimiche (IMC-CNR), Sezione Meccanismi di Reazione, c/o Dipartimento di Chimica, Università degli Studi di Roma "La Sapienza", P.le A. Moro 5, I-00185 Rome, Italy. E-mail: stefano.distefano@uniroma1.it
[b]Area of Genetics and Rare Diseases, Unit of Human Microbiome, Bambino Gesù Children's Hospital, IRCCS, Rome, Italy.

Characterization of complex **4**

[1] H NMR spectrum of complex **1** (for comparison)

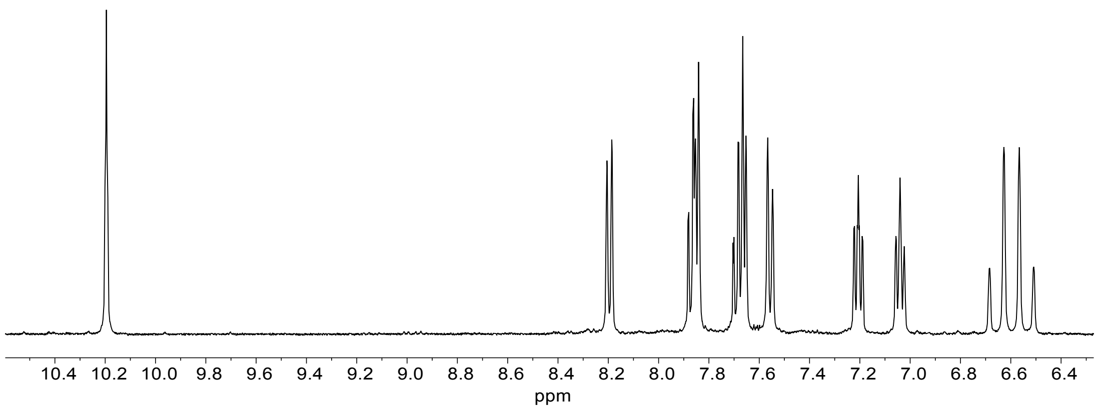

Fig. S1. ^{1}H NMR spectrum (imine and aromatic portions) of complex **1**. From Supporting Information of ref 2b in the main text.

NMR spectra of complex **4**

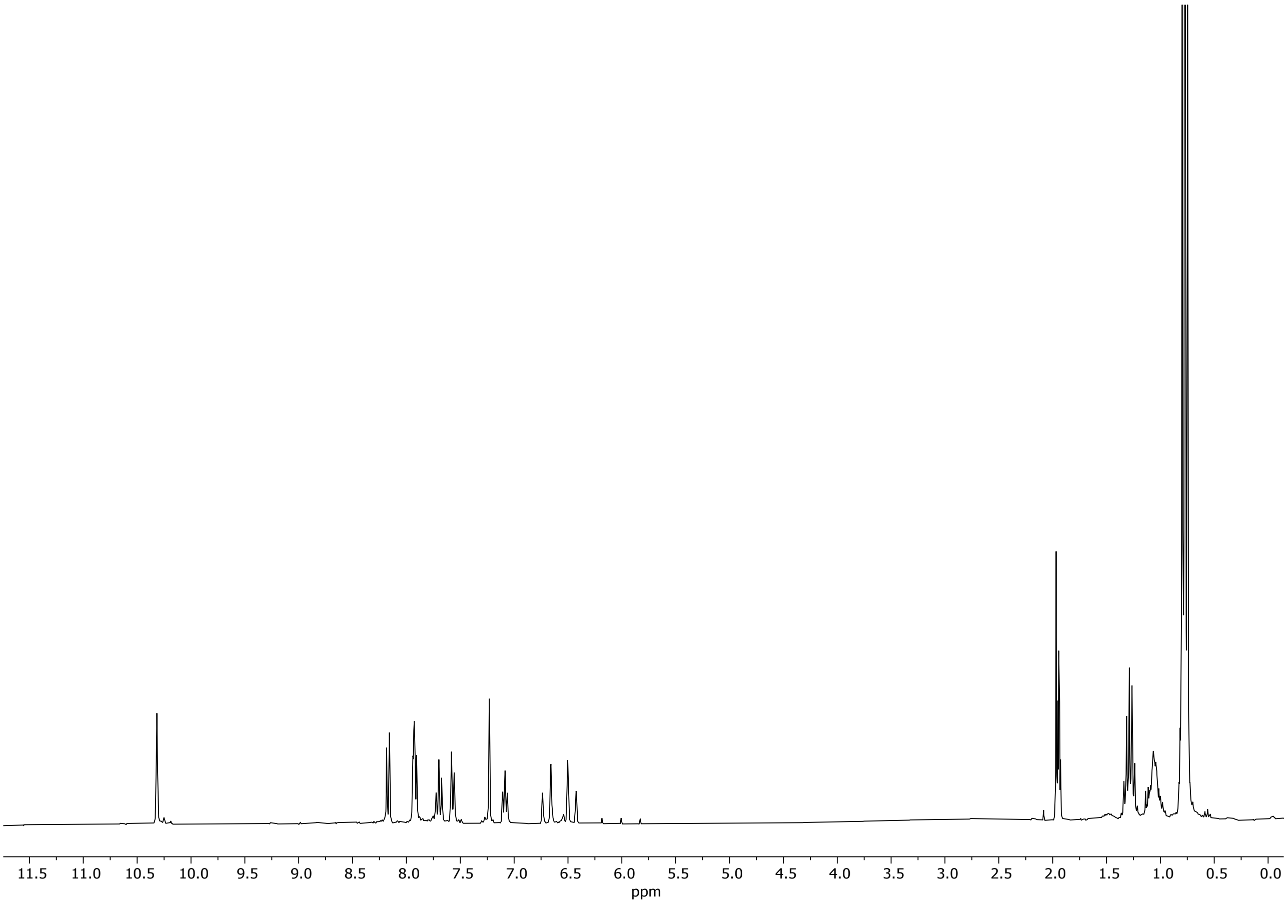

Fig. S2. ^{1}H NMR spectrum of complex **4**. Full spectrum.

^{1}H NMR (300 MHz, CD_3CN) δ 10.32 (s, 2H), 8.17 (d, J = 7.7 Hz, 2H), 7.98 – 7.86 (m, 4H), 7.75 – 7.64 (m, 2H), 7.57 (d, J = 7.8 Hz, 2H), 7.23 (s, 2H), 7.08 (dd, J = 6.4 Hz, 2H), 6.70 (d, J = 23.3 Hz, 2H), 6.46 (d, J = 23.4 Hz, 2H), 1.38 – 1.17 (m, 6H), 1.18 – 0.91 (m, 6H), 0.87 – 0.64 (m, 30H).

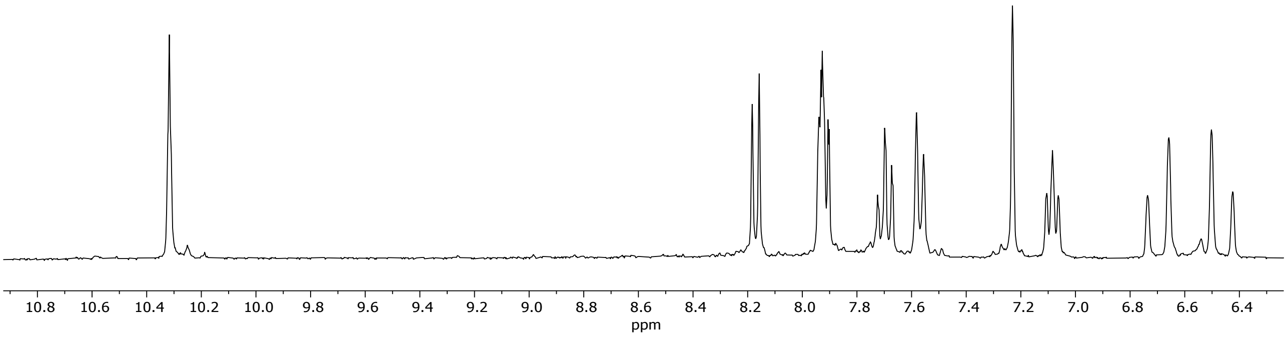

Fig. S3. ^{1}H NMR spectrum of complex **4**. Zoom from 6.4 to 10.8 ppm.

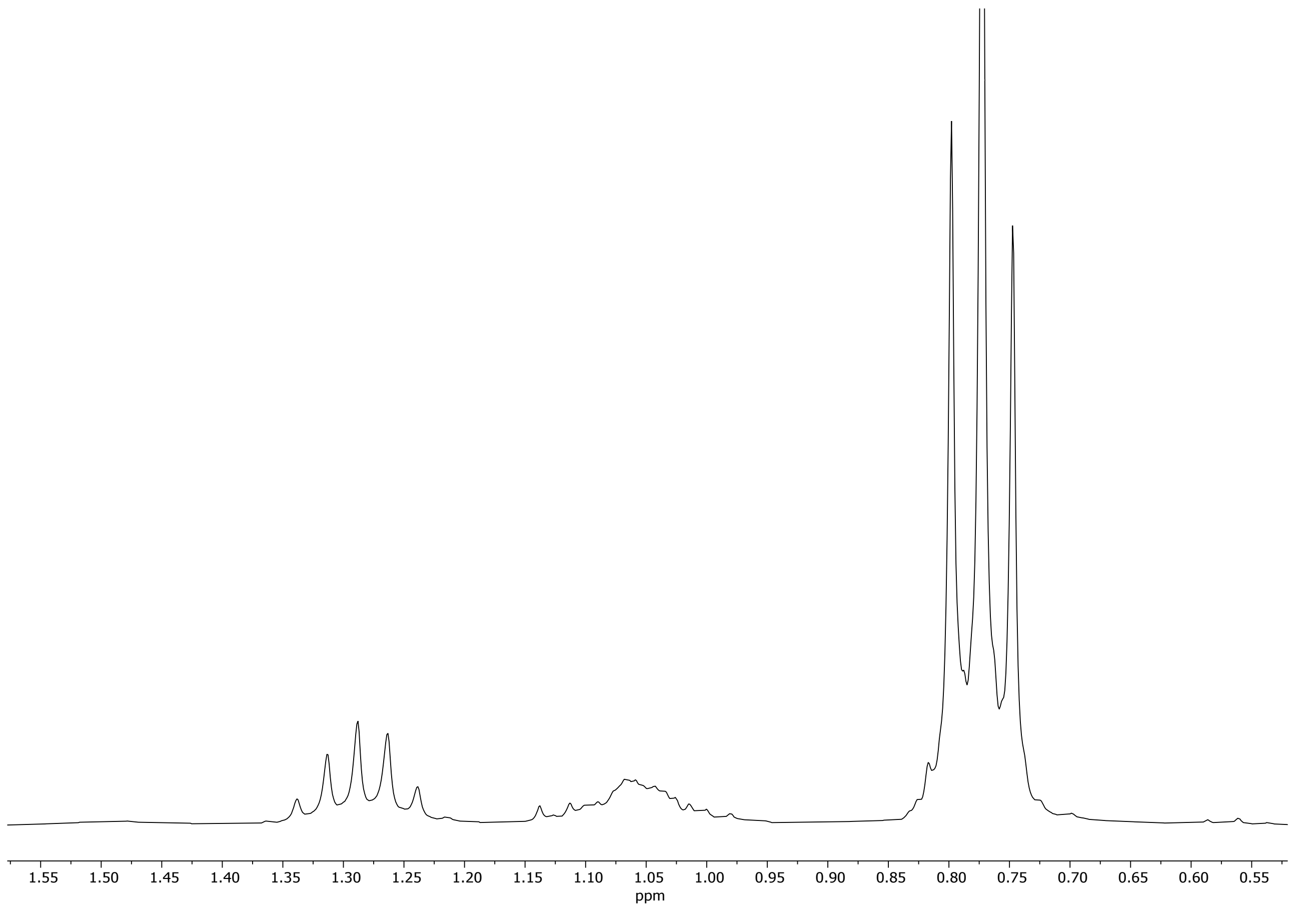

Fig. S4. ^{1}H NMR spectrum of complex **4**. Zoom from 0.55 to 1.55 ppm.

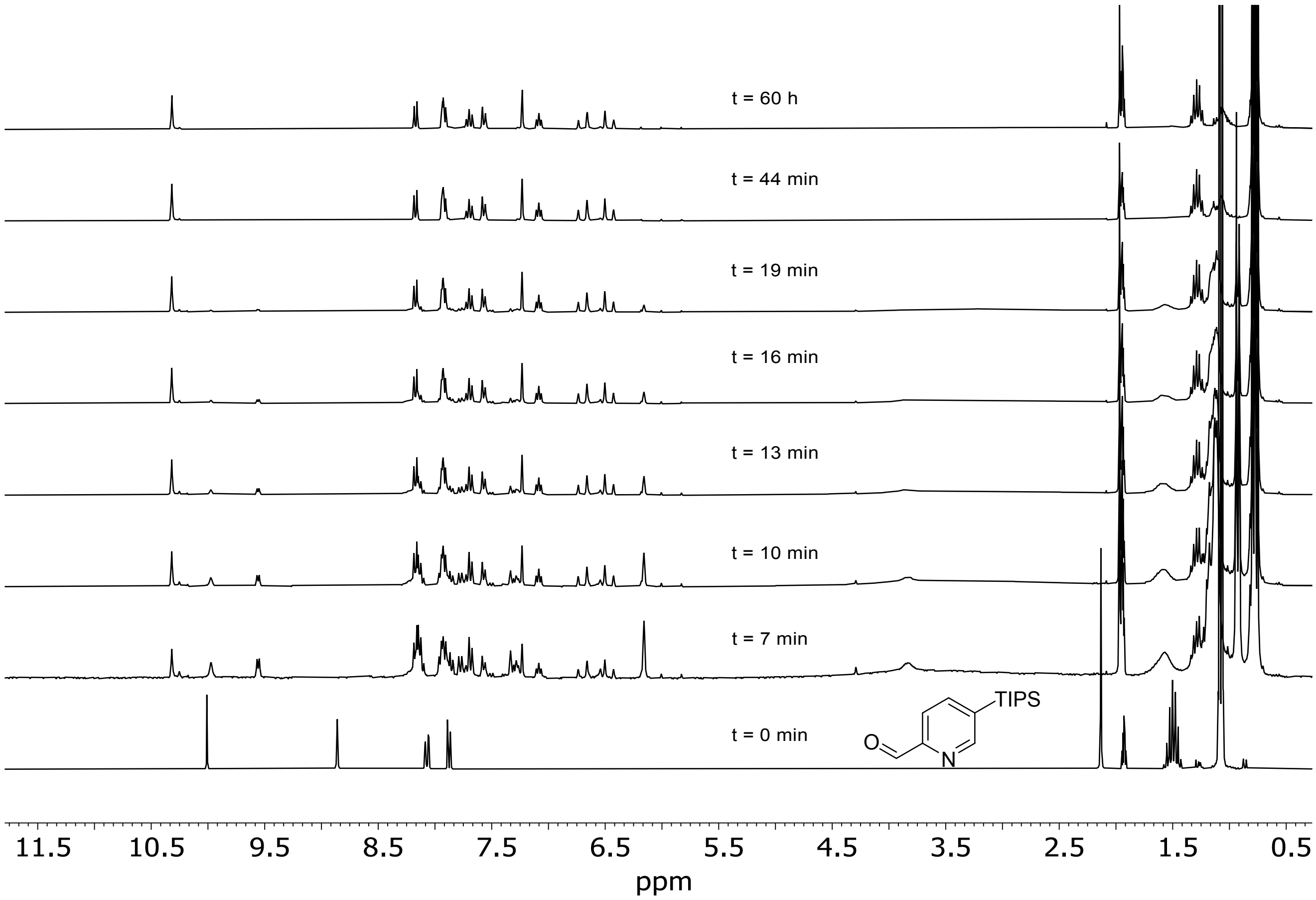

Fig. S5. ^{1}H NMR time monitoring of the self-assembly of 10 mM complex **4** from starting materials $Fe(OTf)_2(CH_3CN)_2$, picolylamine (R' = H) and 5-triisopropylsilyl-pyridine-2-carboxaldehyde (R = $-Si(CH(CH_3)_2)_3$) added in a 1:2:2 ratio, respectively (CD_3CN, 25 °C). At t = 0 min, only 5-triisopropylsilyl-pyridine-2-carboxaldehyde is present. Under these conditions, complex **4** remains unchanged for at least 59 h.

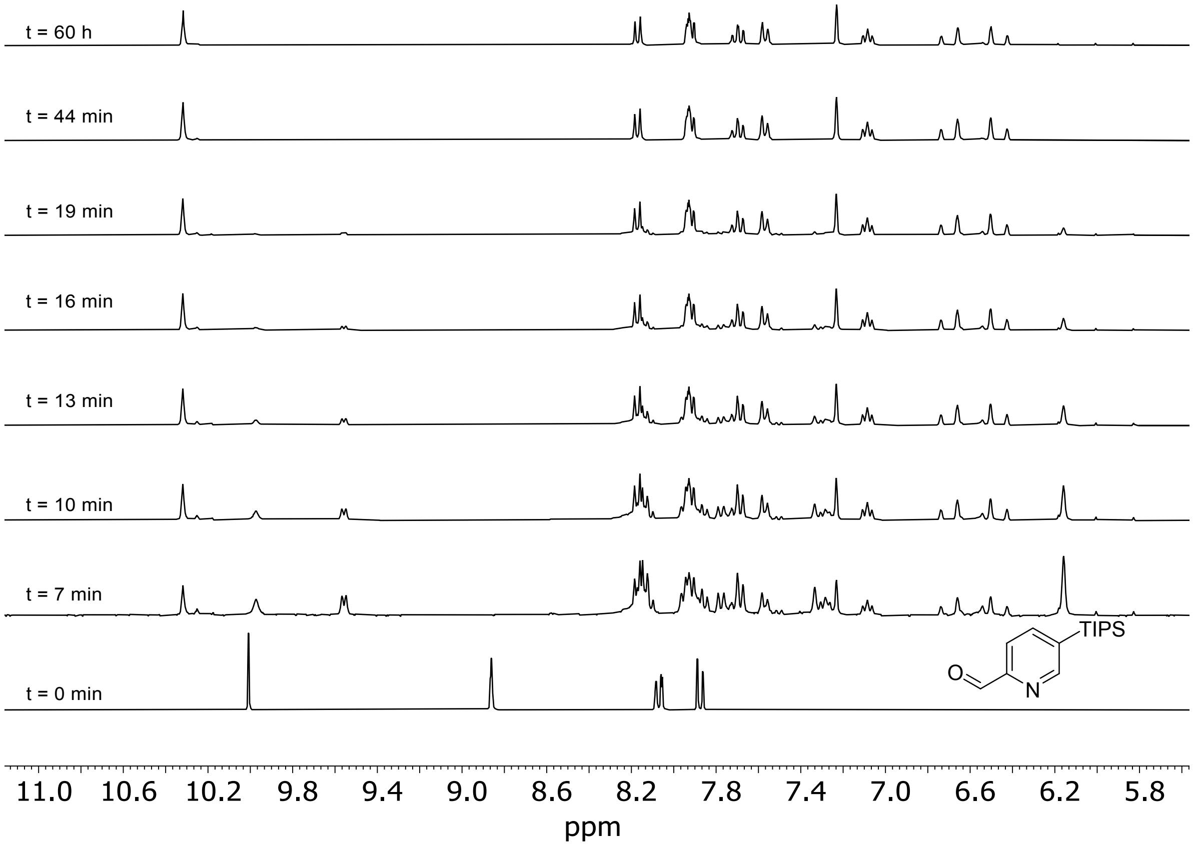

Fig. S6. ^{1}H NMR (zoom from 11 to 5.8 ppm) time monitoring of the self-assembly of 10 mM complex **4** (see Fig. S5 for full spectra) from starting materials $Fe(OTf)_2(CH_3CN)_2$, picolylamine (R' = H) and 5-triisopropylsilyl-pyridine-2-carboxaldehyde (R = $-Si(CH(CH_3)_2)_3$) added in a 1:2:2 ratio, respectively (CD_3CN, 25 °C). At t = 0 min, only 5-triisopropylsilyl-pyridine-2-carboxaldehyde is present. Under these conditions, complex **4** remains unchanged for at least 59 h.

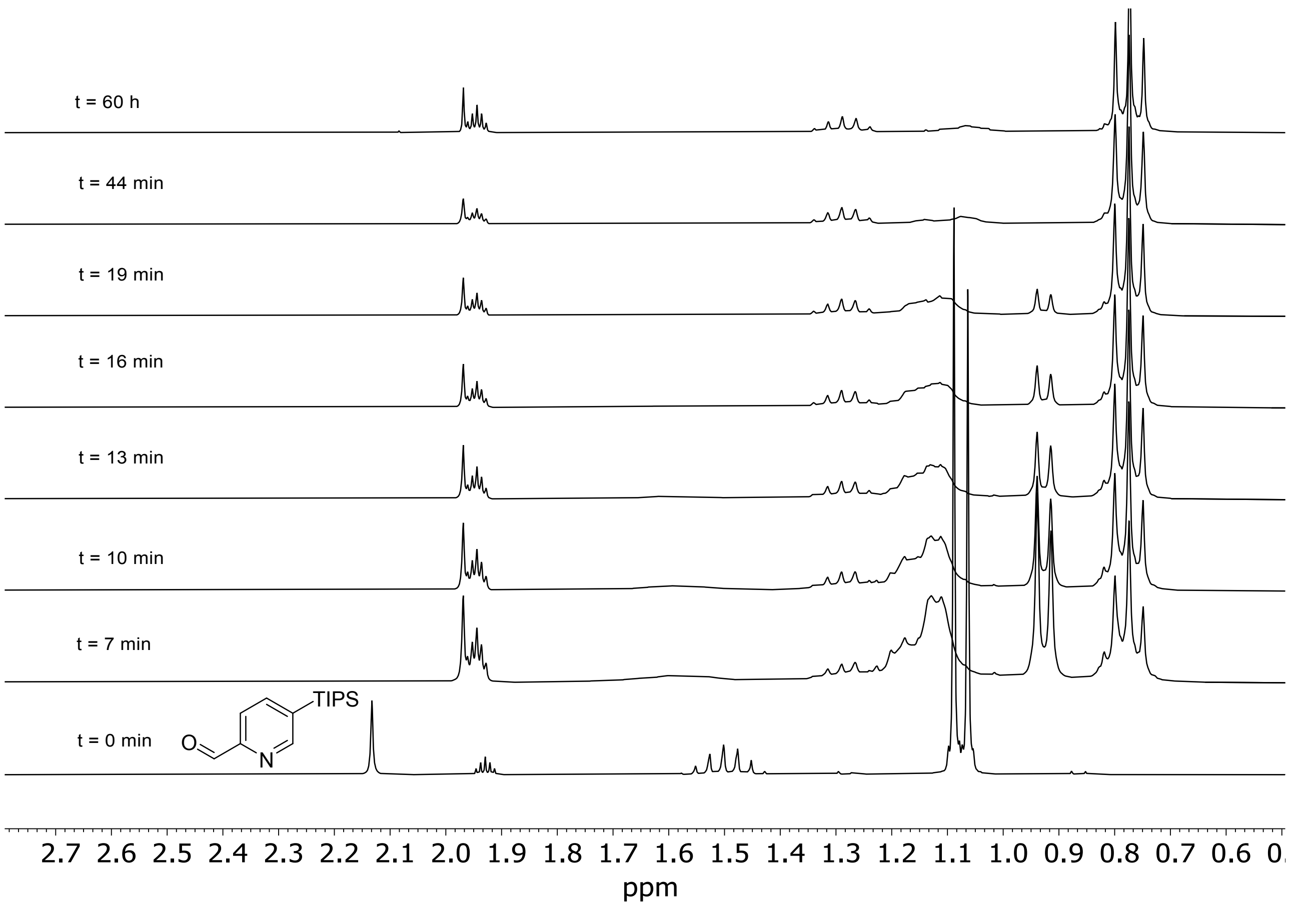

Fig. S7. ^{1}H NMR (zoom from 2.70 to 0.50 ppm) time monitoring of the self-assembly of 10 mM complex **4** (see Fig. S5 for full spectra) from starting materials $Fe(OTf)_2(CH_3CN)_2$, picolylamine (R' = H) and 5-triisopropylsilyl-pyridine-2-carboxaldehyde (R = $-Si(CH(CH_3)_2)_3$) added in a 1:2:2 ratio, respectively (CD_3CN, 25 °C). At t = 0 min, only 5-triisopropylsilyl-pyridine-2-carboxaldehyde is present. Under these conditions, complex **4** remains unchanged for at least 59 h.

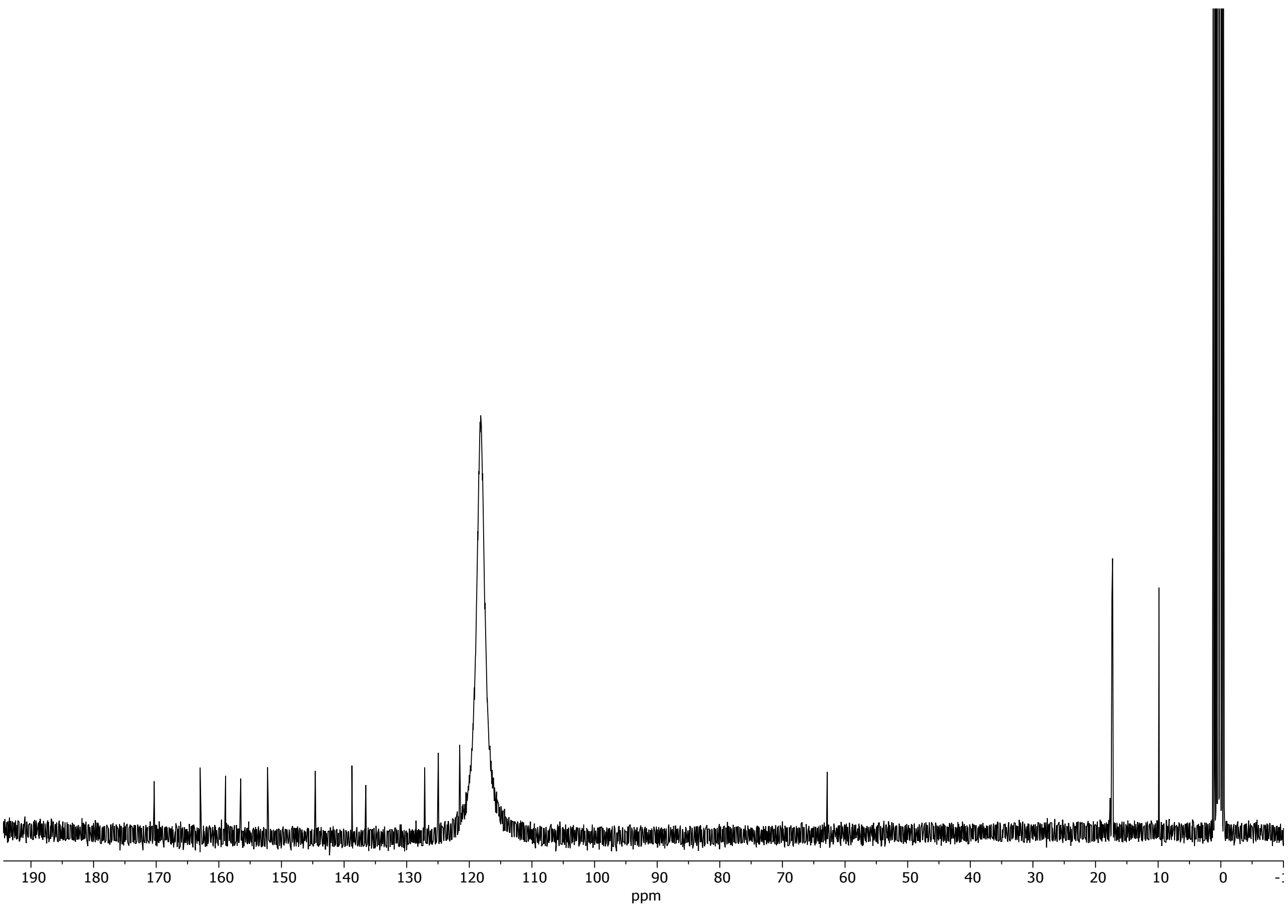

Fig. S8. ^{13}C NMR spectrum of complex **4**.

^{13}C NMR (75 MHz, CD_3CN) δ 170.3, 163.0, 158.9, 156.5, 152.2, 144.6, 138.7, 136.5, 127.1, 125.0, 121.5, 62.9, 17.7, 17.4, 17.3, 9.9.

Signals at 17.7, 17.4 and 17.3 ppm belong to the CH_3 on the triisopropylsilyl groups as it can be inferred from the HSQC experiment (see Fig. S9). The existence of three distinct signals is very likely a consequence of the slowdown of the rotation around the Si-C bond due the increased steric hindrance upon complex formation.

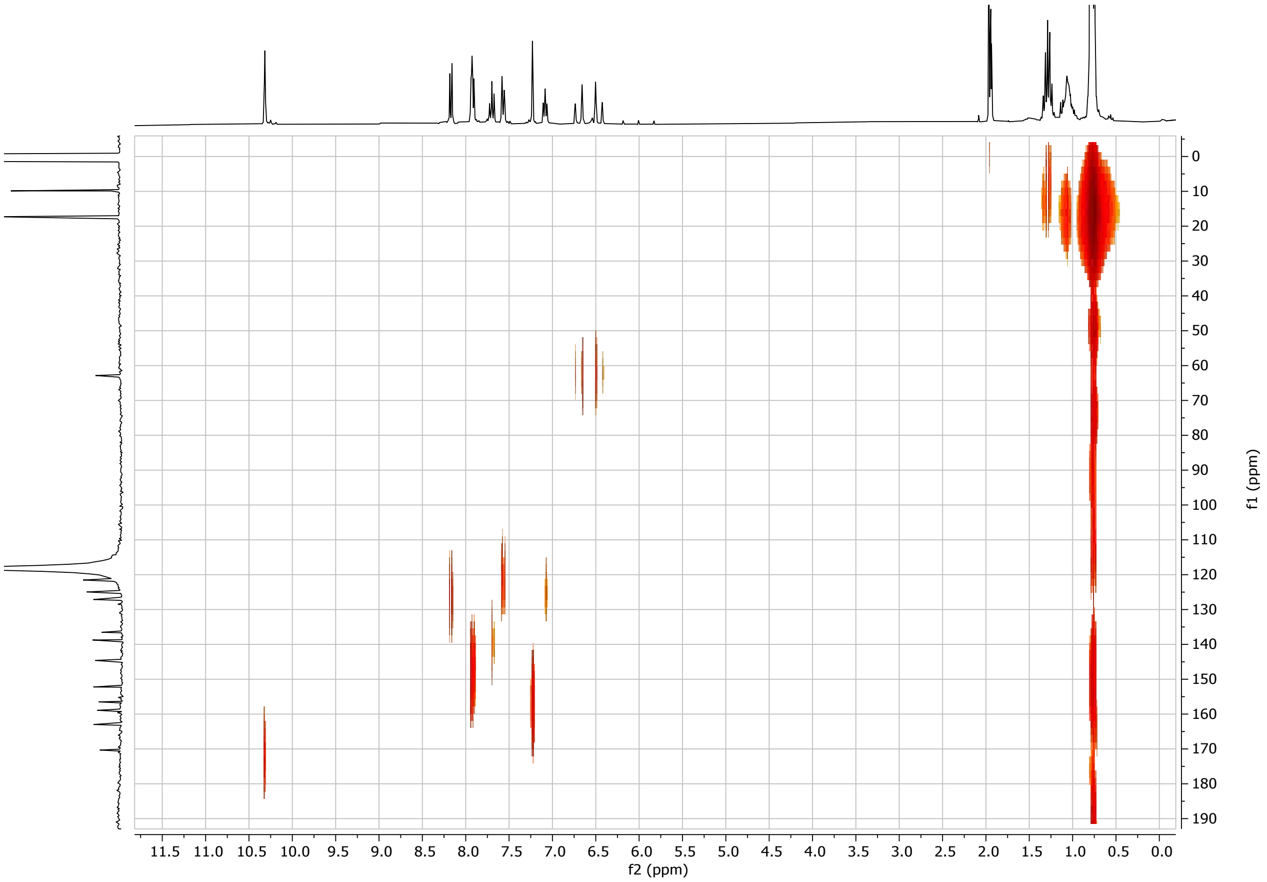

Fig. S9. HSQC spectrum of complex **4**.

UV-Vis absorption spectrum of complex **4**

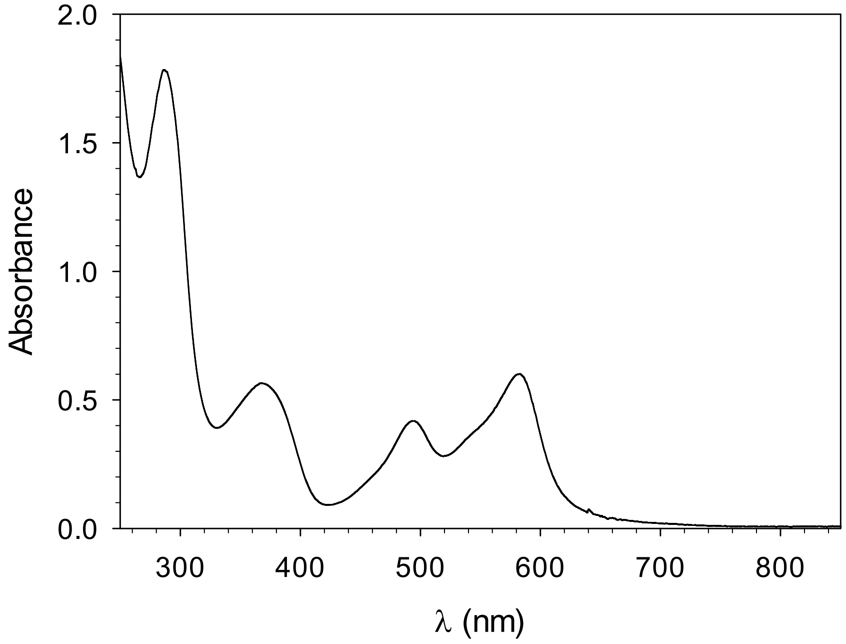

Fig. S10. UV-Vis absorption spectrum of complex **4**; concentration: 8.9×10^{-5} M. Optical path = 1 cm. ε (λ = 494 nm) = 4700 $M^{-1} \times cm^{-1}$, ε (λ = 582 nm) = 6700 $M^{-1} \times cm^{-1}$

Job's plot for complex **4**

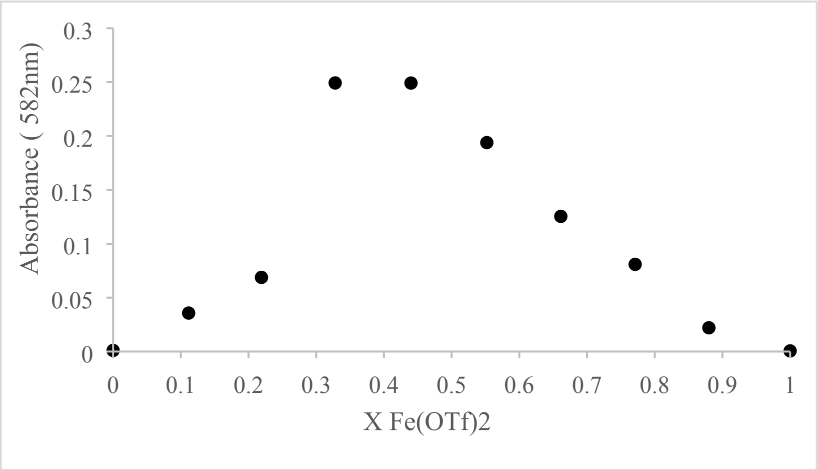

Fig. S11. Job's plot for complex **4**; total concentration [imine ligand] + [$Fe(OTf)_2$] = 1.25×10^{-3} M. Optical path = 1 mm.

Characterization of complex **5**

NMR spectra of complex **5**

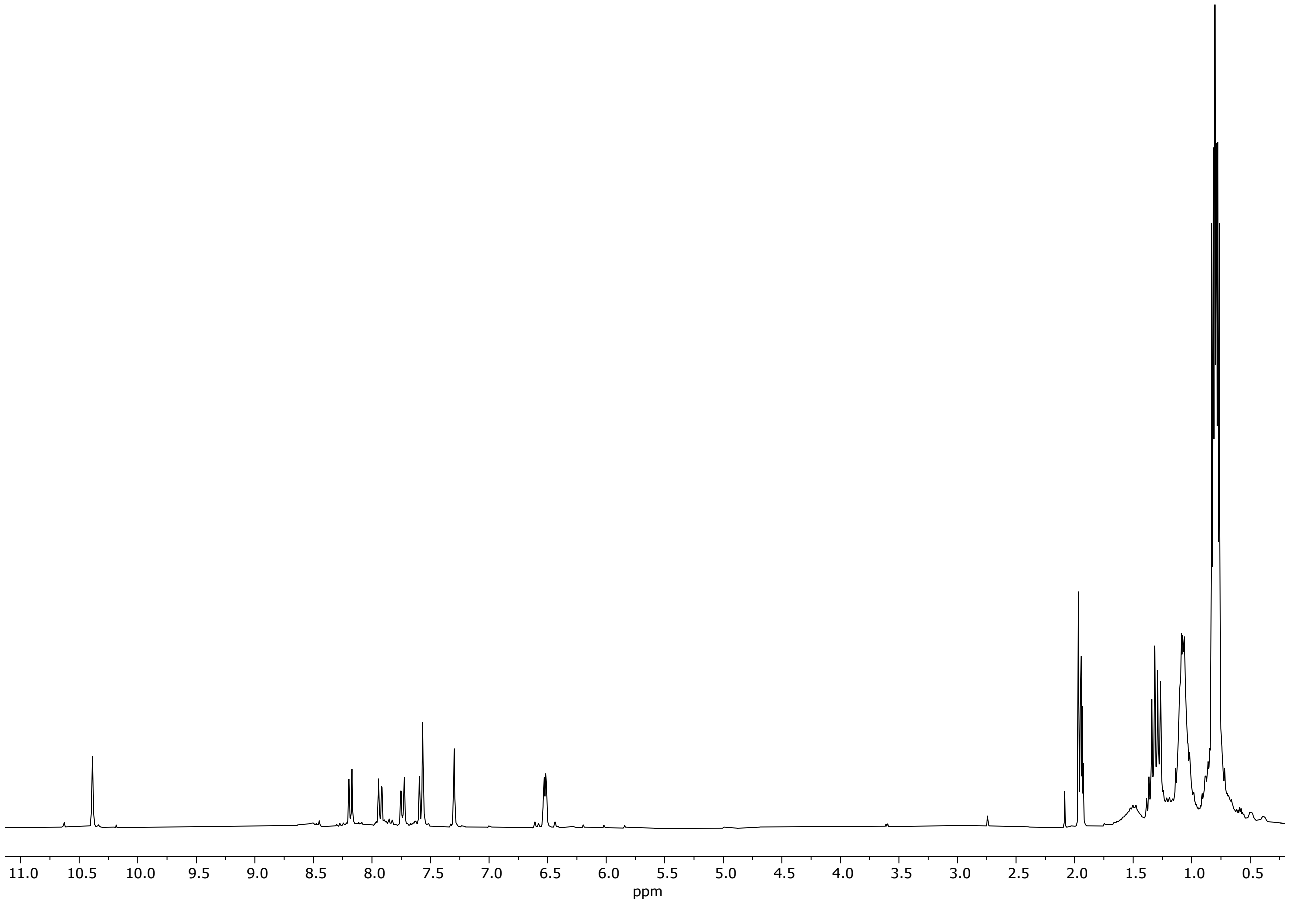

Fig. S12. ^{1}H NMR spectrum of complex **5**. Full spectrum.

^{1}H NMR (300 MHz, CD_3CN) δ 10.39 (s, 2H), 8.18 (d, *J* = 7.7 Hz, 2H), 7.93 (dd, *J* = 7.7, 1.3 Hz, 2H), 7.74 (dd, *J* = 7.8, 1.4 Hz, 2H), 7.58 (d, *J* = 8.3 Hz, 4H), 7.30 (s, 2H), 6.63 – 6.40 (m, 4H), 1.40 – 1.23 (m, 12H), 1.17 – 0.96 (m, 20H), 0.87 – 0.71 (m, 52H).

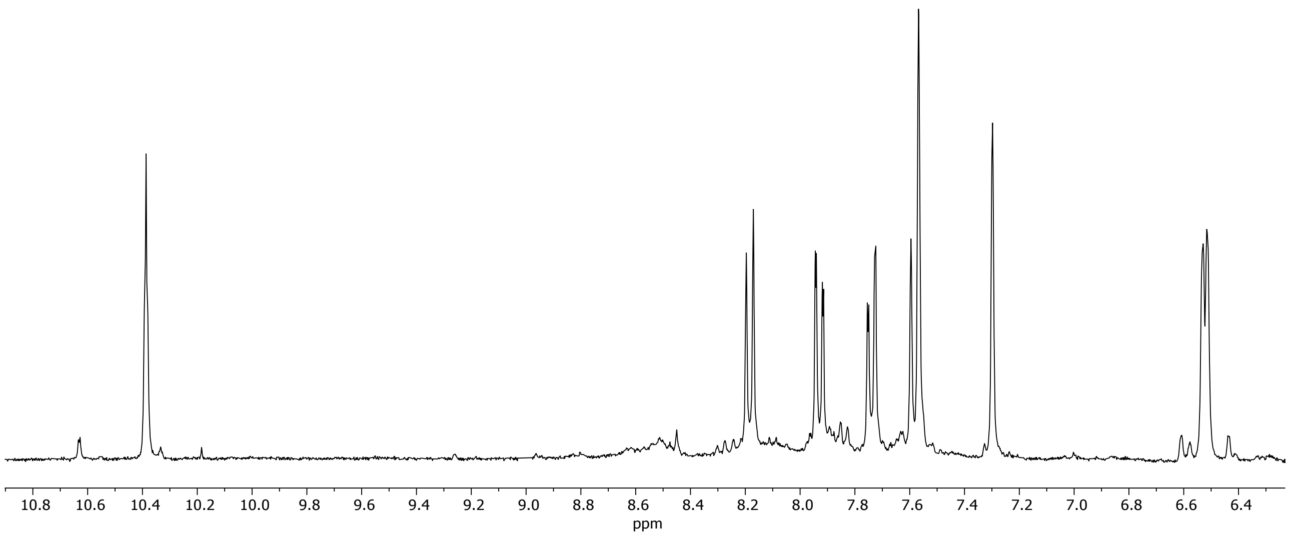

Fig. S13. ^{1}H NMR spectrum of complex **5**. Zoom from 6.4 to 10.8 ppm.

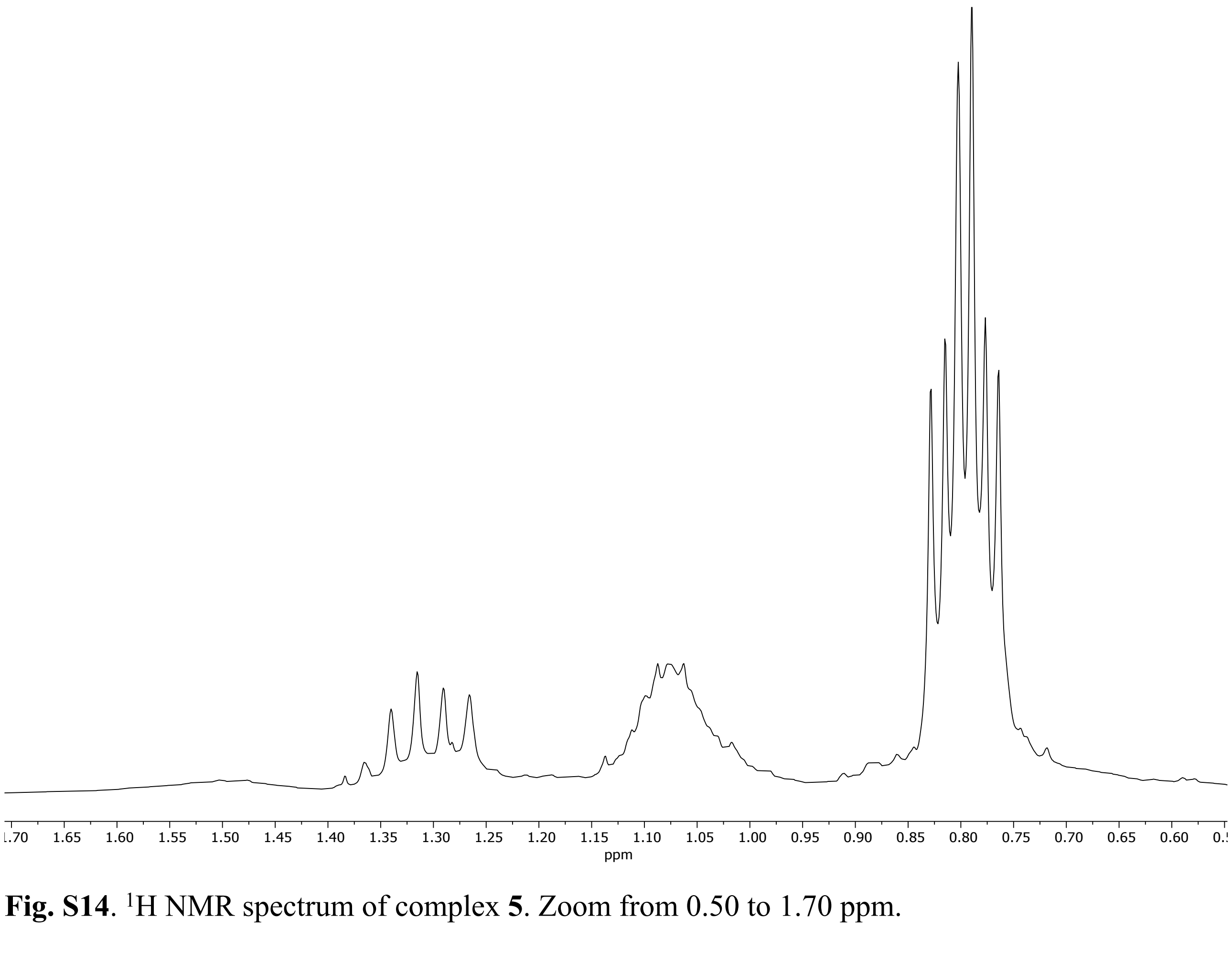

Fig. S14. ^{1}H NMR spectrum of complex **5**. Zoom from 0.50 to 1.70 ppm.

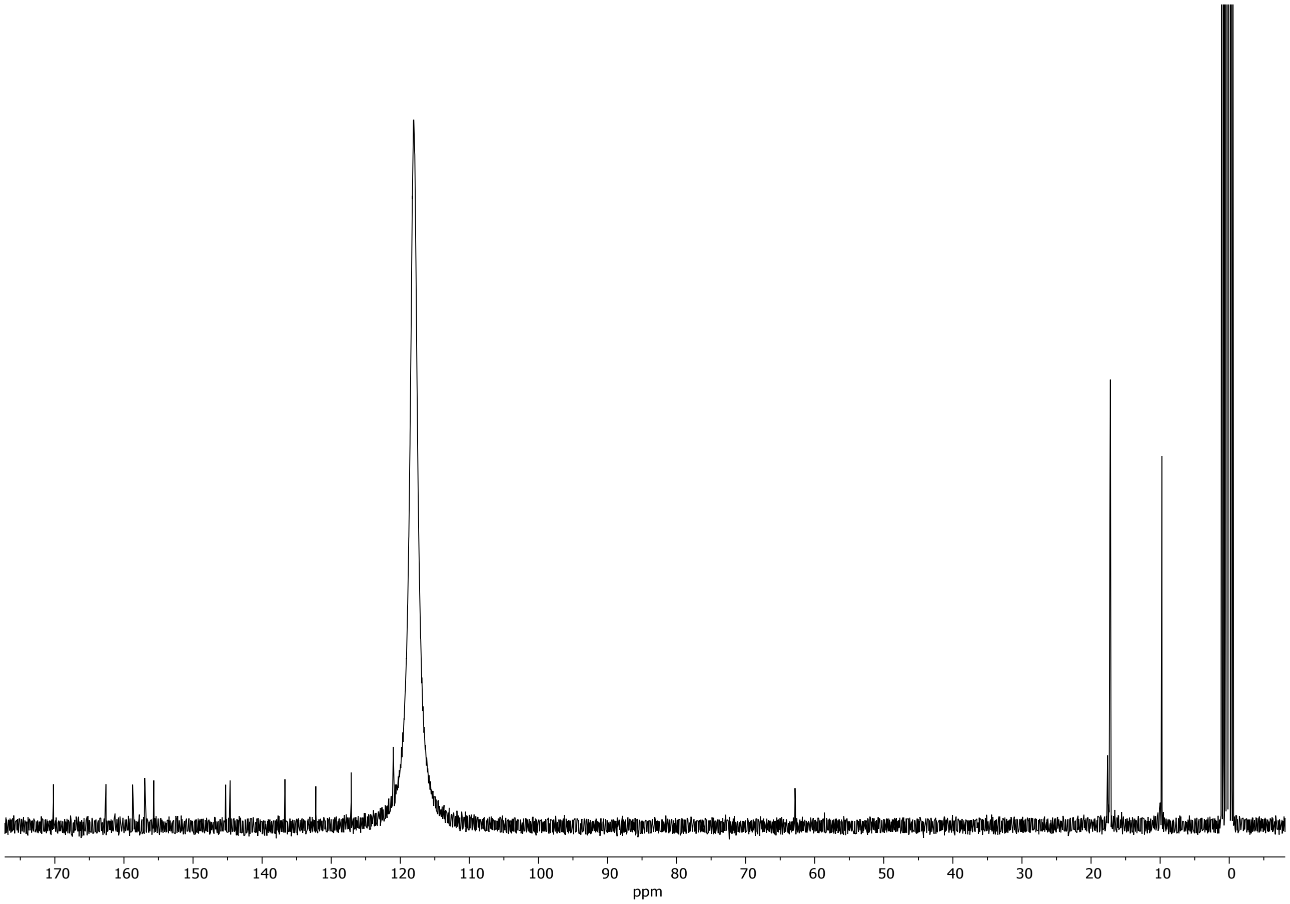

Fig. S15. ^{13}C NMR spectrum of complex **5**.

^{13}C NMR (75 MHz, CD_3CN) δ 170.2, 162.6, 158.7, 157.0, 155.7, 145.3, 144.6, 136.7, 132.2, 127.1, 121.0, 62.9, 17.6, 17.3, 17.2, 9.7.

Signals at 17.6, 17.3 and 17.2 ppm belong to the CH_3 on the triisopropylsilyl groups as it can be inferred from the HSQC experiment (see Fig. S16). The existence of three distinct signals is very likely a consequence of the slowdown of the rotation around the Si-C bond due the increased steric hindrance upon complex formation.

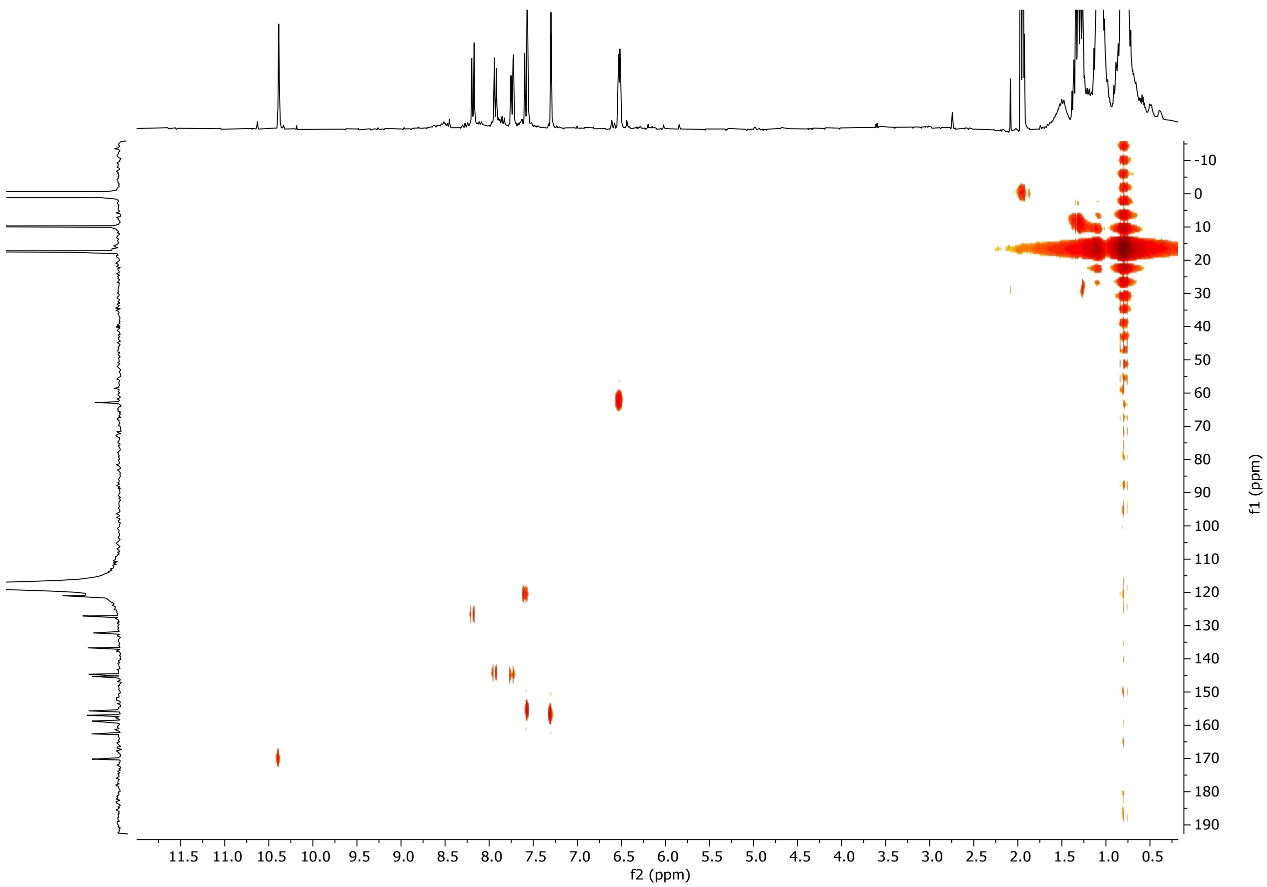

Fig. S16. HSQC spectrum of complex **5**.

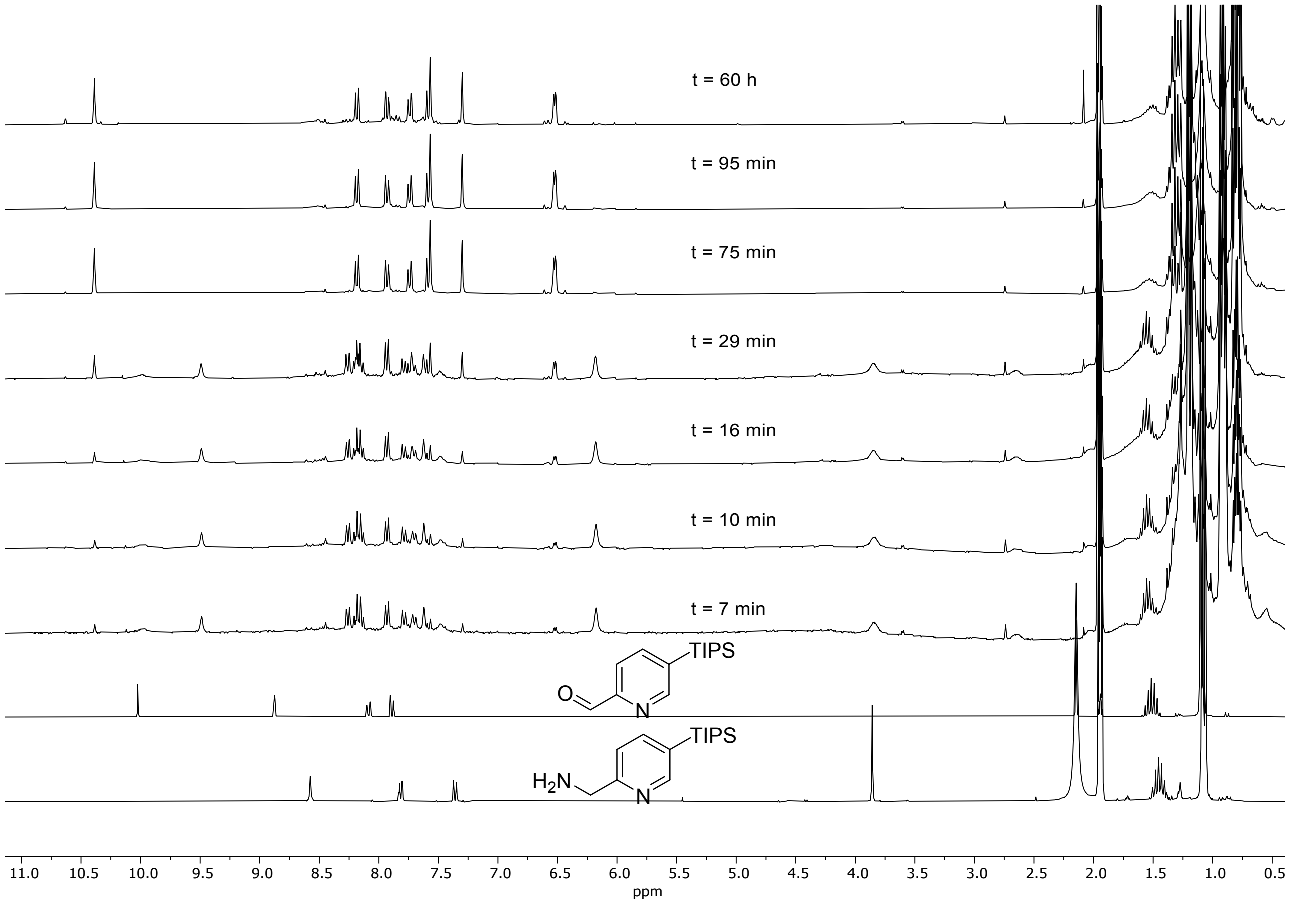

Fig. S17. ^{1}H NMR time monitoring of the self-assembly of 10 mM complex **5** from starting materials $Fe(OTf)_2(CH_3CN)_2$, 5-triisopropylsilylpicolylamine (R' = $-Si(CH(CH_3)_2)_3$) and 5-triisopropylsilyl-pyridine-2-carboxaldehyde (R = $-Si(CH(CH_3)_2)_3$) added in a 1:2:2 ratio, respectively (CD_3CN, 25 °C). At t = 0 min, only 5-triisopropylsilyl-pyridine-2-carboxaldehyde is present. Under these conditions, complex **5** remains unchanged for at least 59 h.

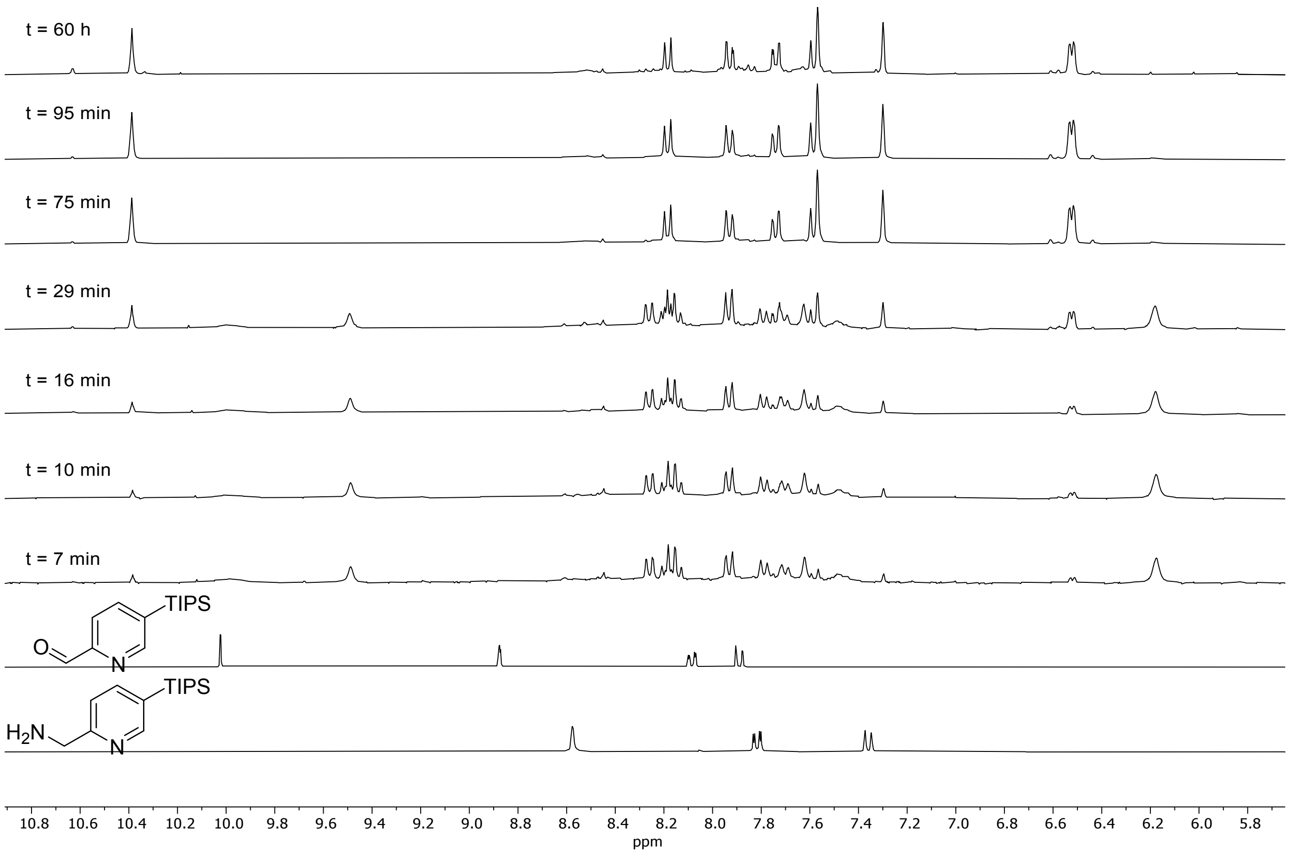

Fig. S18. ^{1}H NMR time monitoring (Zoom from 5.80 to 10.80 ppm) of the self-assembly of 10 mM complex **5** from starting materials $Fe(OTf)_2(CH_3CN)_2$, 5-triisopropylsilylpicolylamine (R' = $-Si(CH(CH_3)_2)_3$) and 5-triisopropylsilyl-pyridine-2-carboxaldehyde (R = $-Si(CH(CH_3)_2)_3$) added in a 1:2:2 ratio, respectively (CD_3CN, 25 °C). At t = 0 min, only 5-triisopropylsilyl-pyridine-2-carboxaldehyde is present. Under these conditions, complex **5** remains unchanged for at least 59 h.

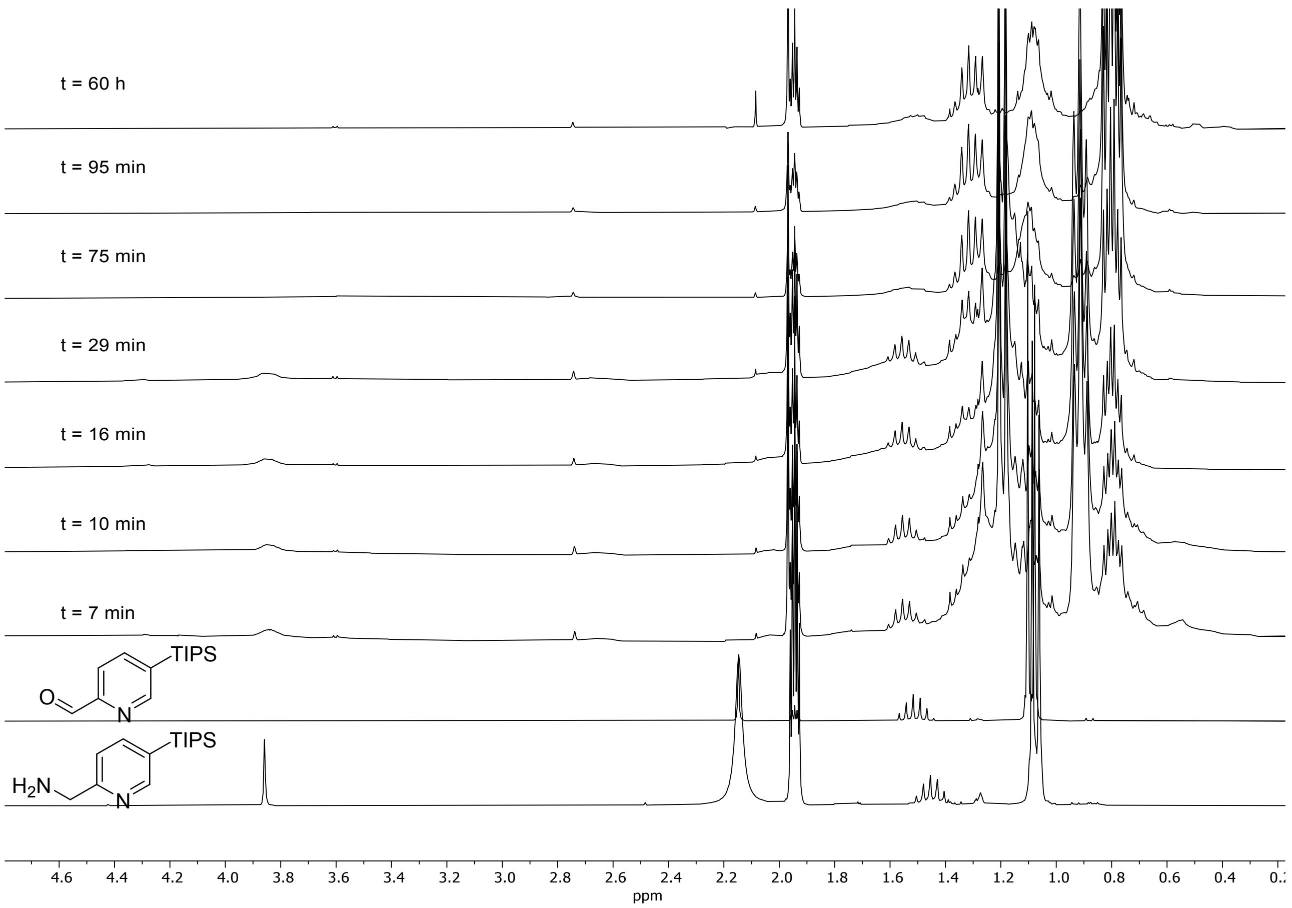

Fig. S19. ^{1}H NMR time monitoring (zoom from 4.60 to 0.30 ppm) of the self-assembly of 10 mM complex **5** from starting materials $Fe(OTf)_2(CH_3CN)_2$, 5-triisopropylsilylpicolylamine (R' = $-Si(CH(CH_3)_2)_3$) and 5-triisopropylsilyl-pyridine-2-carboxaldehyde (R = $-Si(CH(CH_3)_2)_3$) added in a 1:2:2 ratio, respectively (CD_3CN, 25 °C). At t = 0 min, only 5-triisopropylsilyl-pyridine-2-carboxaldehyde is present. Under these conditions, complex **5** remains unchanged for at least 59 h.

UV-Vis absorption spectrum of complex **5**

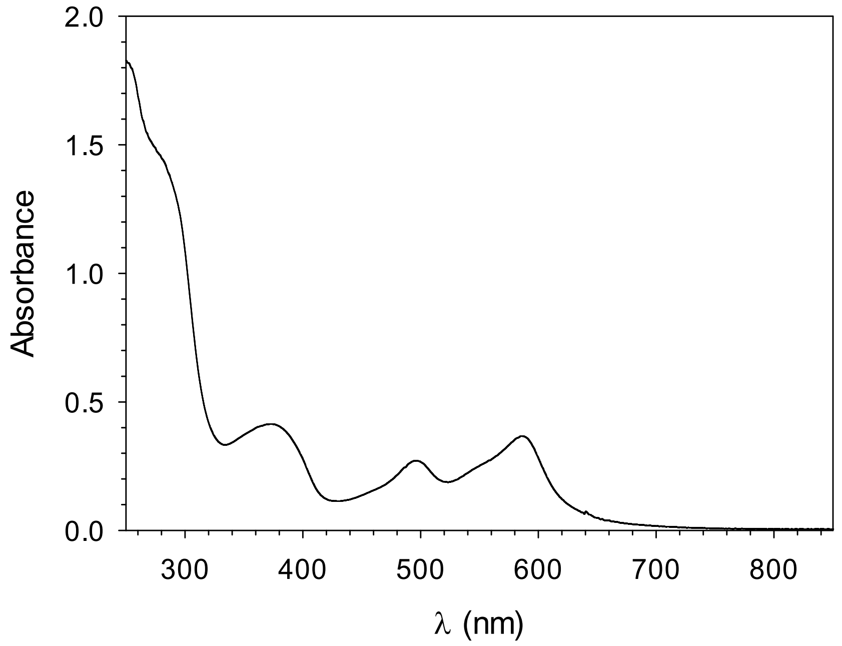

Fig. S20. UV-Vis absorption spectrum of complex **5**; concentration: 7.0×10^{-5} M. Optical path = 1 cm. ε (λ = 498 nm) = 3900 $M^{-1} \times cm^{-1}$, ε (λ = 587 nm) = 5300 $M^{-1} \times cm^{-1}$

Job's plot for complex **5**

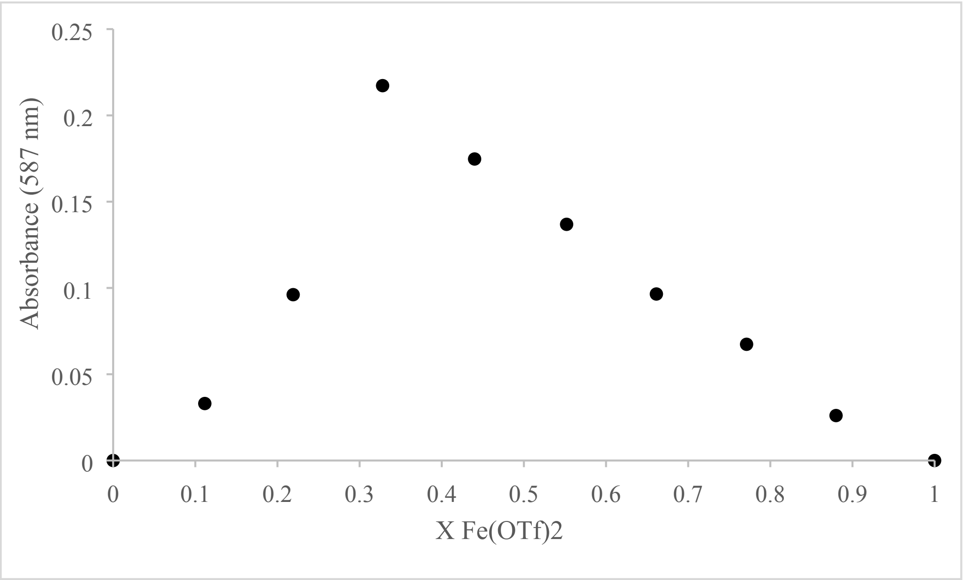

Fig. S21. Job's plot for complex **5**; total concentration [imine ligand] + $[Fe(OTf)_2]$ = 1.25×10^{-3} M. Optical path = 1 mm.

UV-Vis absorption spectrum of complexes **1**, **4** and **5**

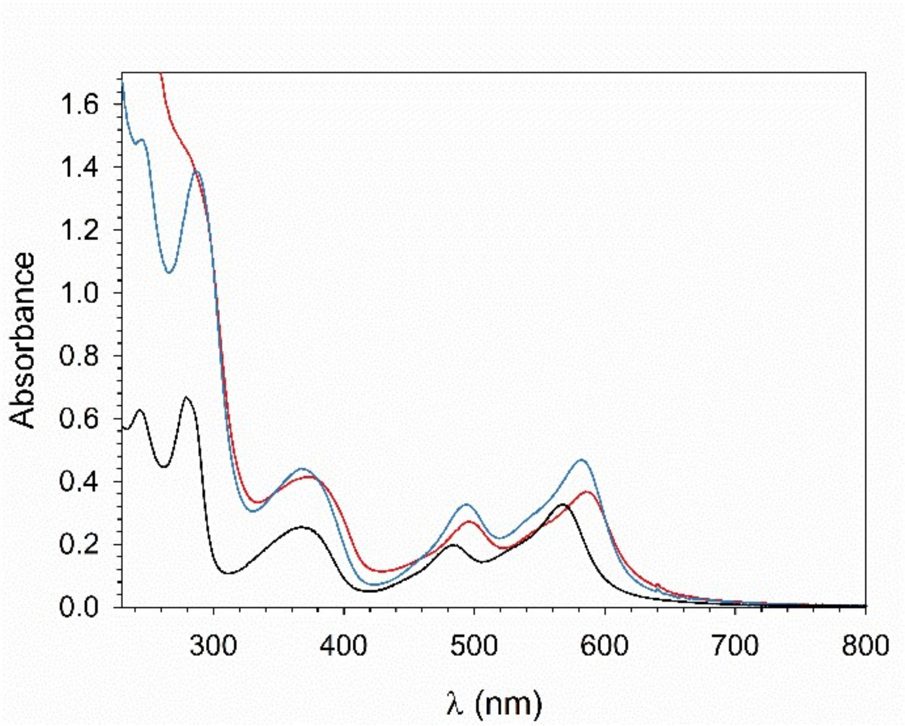

Fig. S22. UV-Vis spectra of complexes **1** (black), **4** (blue) and **5** (red) (CH_3CN, 25 °C, 0.070 mM, optical path 1cm).

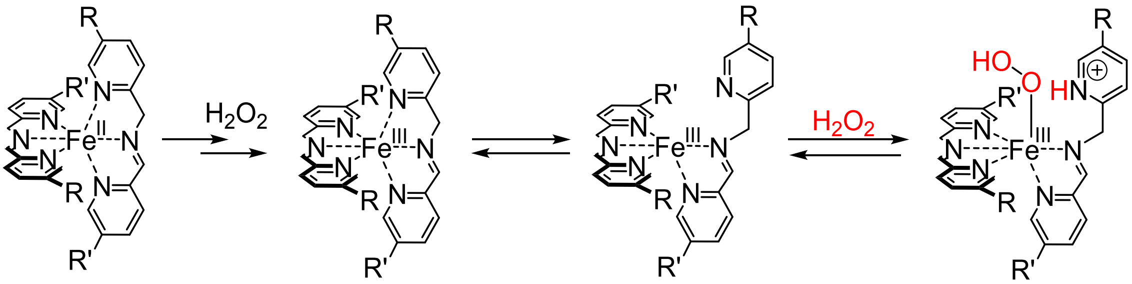

Fig. S23. Proposed mechanism for activation of H_2O_2 by complexes **1**, **4** and **5**.
